# Orientational mapping of minerals in Pierre shale using X-ray diffraction tensor tomography

**DOI:** 10.1107/S205225252100587X

**Published:** 2021-07-17

**Authors:** Fredrik K. Mürer, Aldritt Scaria Madathiparambil, Kim Robert Tekseth, Marco Di Michiel, Pierre Cerasi, Basab Chattopadhyay, Dag W. Breiby

**Affiliations:** a PoreLab, Department of Physics, Norwegian University of Science and Technology, Høgskoleringen 5, Trondheim 7491, Norway; b ESRF, The European Synchrotron, 71 Avenue des Martyrs, Grenoble 38000, France; c Petroleum Department, SINTEF Industry, Trondheim 7465, Norway; d Department of Microsystems, University of South-Eastern Norway, Campus Vestfold, Borre 3184, Norway

**Keywords:** X-ray diffraction, shales, mineralogy, orientation mapping, inorganic materials, high-density inclusions, computed tomography

## Abstract

Shales have a complex mineralogy with structural features spanning several length scales, making them notoriously difficult to fully understand. Conventional attenuation-based X-ray computed tomography (CT) measures density differences, which, owing to the heterogeneity and sub-resolution features in shales, makes reliable interpretation of shale images a challenging task. CT based on X-ray diffraction (XRD-CT), rather than intensity attenuation, is becoming a well established technique for non-destructive 3D imaging, and is especially suited for heterogeneous and hierarchical materials. XRD patterns contain information about the mineral crystal structure, and crucially also crystallite orientation. Here, we report on the use of orientational imaging using XRD-CT to study crystallite-orientation distributions in a sample of Pierre shale. Diffraction-contrast CT data for a shale sample measured with its bedding-plane normal aligned parallel to a single tomographic axis perpendicular to the incoming X-ray beam are discussed, and the spatial density and orientation distribution of clay minerals in the sample are described. Finally, the scattering properties of highly attenuating inclusions in the shale bulk are studied, which are identified to contain pyrite and clinochlore. A path forward is then outlined for systematically improving the structural description of shales.

## Abstract 2

X-ray diffraction tensor tomography is used to map clay mineral orientation and high-density inclusions in a sample of Pierre shale.

## 1. Introduction   

The orientation of nano-crystallites affects the macroscopic physical properties in a wide range of hierarchical materials such as bones (Stock, 2015 ▸), polymers (Baer *et al.*, 1987 ▸) and shales (Leu *et al.*, 2016 ▸). X-ray micro-computed tomography (µCT), based on intensity attenuation of the beam as it propagates through the specimen, has, owing to technical advances during the last decade, become a workhorse for studies of complex natural and manmade material structures, both at home laboratories and at synchrotrons. However, despite its successes, µCT has some important limitations. Using µCT, the structural information provided is usually in the micrometre range and it is difficult to distinguish materials of similar electron density, thus limiting the information obtained about the underlying nature of the material. For example, with unresolved nanoscale porosity, the effective attenuation of a volume element in the specimen can take a wide range of values, making the segmentation and subsequent analysis prone to error. While electron microscopies can give superior structural resolution for certain samples, the field of view is vanishingly small, with the associated risk of rendering the analysis of little macroscale relevance. For all these reasons, it is of high importance to develop new methods that can be used at different length scales and inherently contain structural information arising at the nanoscale.

Wide-angle X-ray diffraction (XRD) is a firmly established technique for crystallographic studies, giving conclusive information about the atomic scale arrangements. Owing to recent technical advances, perhaps most notably fast-readout area detectors (Vaughan *et al.*, 2020 ▸), various scanning XRD techniques have been developed, including hierarchical orientational mapping (Paris, 2008 ▸), whereby the averaged orientation properties of molecular structures at the Ångström length scale are probed over larger length scales ranging from micrometres to several millimetres. For fine-grained isotropic materials, X-ray diffraction computed tomography (XRD-CT) (Harding *et al.*, 1987 ▸; Kleuker *et al.*, 1998 ▸; Stock *et al.*, 2008 ▸; Mürer *et al.*, 2018 ▸; Grünewald *et al.*, 2020 ▸) currently allows 3D non-destructive determination of material composition, particle size, shape and crystal lattice parameters of millimetre-sized samples with spatial resolution typically in the 10^−1^–10^2^ micrometre range (Birkbak *et al.*, 2015 ▸; Palle *et al.*, 2020 ▸). Reconstructed diffractograms can be combined with Rietveld refinement (Rietveld, 1969 ▸; Frølich *et al.*, 2016 ▸) or Williamson–Hall analysis (Williamson & Hall, 1953 ▸; Mürer *et al.*, 2021 ▸) for accurate determination of material volume fractions, and hence be used to obtain accurate spatially resolved maps of sample composition, crystallite size and strain. XRD-CT normally requires that the specimen materials are fine grained and isotropic, ensuring that the scattering signal from each sample voxel is rotationally invariant (Feldkamp *et al.*, 2009 ▸). Qualitative material-distinguishing XRD-CT can still be performed if the materials are only weakly anisotropic by suppressing the orientation information contained in the diffraction patterns during the tomographic reconstruction (Stock *et al.*, 2008 ▸; Mürer *et al.*, 2018 ▸). Moreover, scattering approximately parallel to the rotation axis is readily seen to be invariant with respect to sample rotation, allowing moderately complex orientation arrangements to be reconstructed (Egan *et al.*, 2013 ▸; Gürsoy *et al.*, 2015 ▸; Mürer *et al.*, 2018 ▸) using *e.g.* the standard filtered backprojection (FBP) algorithm. Small-angle X-ray scattering CT (Schroer *et al.*, 2006 ▸; Feldkamp *et al.*, 2009 ▸) utilizes similar working principles as XRD-CT and is used to probe larger structural features in the tens of nanometres range.

Whereas XRD has been used for sample-averaged texture measurements for decades, small-angle scattering and X-ray diffraction tensor tomography (SASTT, XRDTT) have been recently introduced (Skjønsfjell *et al.*, 2016 ▸; Liebi *et al.*, 2015 ▸, 2018 ▸) as techniques to retrieve the spatially resolved 3D nanostructure *orientation* distributions across millimetre-sized samples. SASTT and XRDTT utilize a similar experimental setup as XRD-CT, however the sample is rotated about two orthogonal axes during the experiment to allow sufficient sampling of the direction-dependent scattering. Orientation information can also be retrieved with XRDTT by using a single tomography axis if the measured scattering is predominantly in the direction of the tomography axis. A model function based on spherical harmonics (Roe & Krigbaum, 1964 ▸) can be used to describe the orientational distribution function for each sample voxel, typically of size (1–100 µm)^3^, and this model function is fitted to the measured data using *e.g.* a conjugated gradient optimization routine. While traditional pole figure (texture) analysis aims to describe the averaged orientation distribution function valid for a presumably uniform sample (Bunge, 1969 ▸; Breiby & Samuelsen, 2003 ▸), tensor tomography can reconstruct 3D maps of the variations of the texture in heterogeneous non-uniform samples. SASTT and XRDTT have recently been demonstrated on bone (Liebi *et al.*, 2015 ▸, 2018 ▸; Guizar-Sicairos *et al.*, 2020 ▸; Mürer *et al.*, 2021 ▸), polymers (Skjønsfjell *et al.*, 2016 ▸) and brain tissue (Gao *et al.*, 2019 ▸). While SASTT/XRDTT have facilitated 3D orientational X-ray imaging, the experiments are time consuming because many (typically >10^6^) diffraction patterns have to be collected to adequately probe the sample. Previous studies report measurement times of >24 h when the sampled volume is ∼50^3^ voxels. In addition to being time consuming, the tensor-tomography experiments expose the samples to high radiation doses, potentially causing radiation damage. Therefore, ways of reducing the measurement time and dose in tensor-tomography experiments need to be further investigated.

Shales are fine-grained sedimentary rocks and exist in a variety of types with varying chemical composition (Shaw & Weaver, 1965 ▸). Shales contain several clay mineral phases, mixed with silt-sized particles of other minerals, commonly quartz and calcite. The oriented clay phases, consisting typically of flake-shaped grains exhibiting preferred orientations relative to the bedding planes, give rise to anisotropic scattering in both the small- and wide-angle regimes (Wenk *et al.*, 2010 ▸). Shales show pronounced fissility, *i.e.* a tendency of splitting along flat planes parallel to the stratification. Shales typically split into thin laminae of a few millimetres thickness, reflecting the parallel orientation of clay mineral flakes. Sandstones, with their simpler composition and high porosity (10–40%), often exhibit a rather uniform pore-size distribution, and are currently considered well understood in terms of both mechanical properties and fluid permeability (Keelan, 1982 ▸). Shales, on the other hand, with numerous mineral phases present, combined with nanoscale porosity and pronounced anisotropy, await scientific breakthroughs with improved imaging methods before a better understanding can be reached (Ma *et al.*, 2017 ▸).

In this article, we demonstrate XRDTT of a sample of Pierre shale. First, we present the mineral composition and the observed texture in the diffraction patterns, which can be used to infer the orientation of clay minerals. Thereafter, we discuss the 3D orientation of the clay minerals present in the sample volume and demonstrate the feasibility of using XRDTT for retrieving the sample nanostructure orientation by using a tomography setup with a single sample rotation axis. Finally, we study the scattering from comparably large high-density mineral grains in the shale sample.

## 2. Experimental   

### 2.1. Sample   

The Pierre shale sample was extracted from blocks obtained from a Colorado quarry (Cerasi *et al.*, 2017 ▸), and further cored to make cylindrical plugs from which fragments were detached and stored in air at room temperature. The sample was glued onto a pin and mounted on a goniometer head with its bedding-plane normal essentially co-aligned with the tomographic rotation axis.

### 2.2. XRD-CT   

#### 2.2.1. CT measurements   

**Figure 1:**
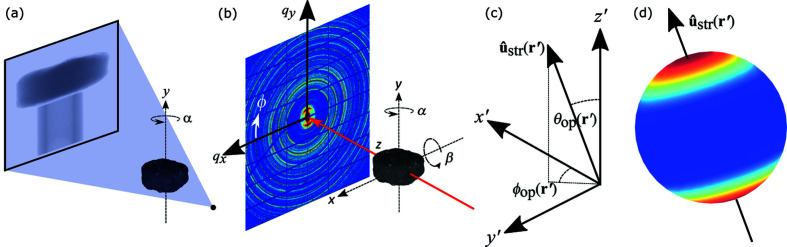
Schematic setups for (*a*) home-laboratory cone-beam attenuation-contrast CT and (*b*) synchrotron parallel-beam XRD-CT. (*c*) A sample coordinate system **r**′ = (*x*′, *y*′, *z*′)^T^ defined such that the *z*′ axis is parallel to the laboratory frame *y* axis. The local preferred orientation for each sample voxel is described by **û**_str_(**r**′). (*d*) An illustration of a uniaxial scattering intensity distribution for a single sample voxel.

The very same Pierre shale sample was measured using both home-laboratory attenuation-contrast µCT and synchrotron XRD-CT, see Fig. 1 ▸ for sketches of the setups and coordinate conventions. Studying the same sample with both modalities allows the diffraction-contrast 3D tomograms to be co-registered and quantitatively compared with the higher-resolution µCT 3D tomogram.

XRD-CT measurements were performed at the beamline ID15A (Vaughan *et al.*, 2020 ▸) at the ESRF, Grenoble, France. A partly coherent beam of photon energy 50.00 keV was used (λ = 0.2480 Å). The beam was collimated into a pencil-shaped beam of dimensions 50 × 50 µm by using compound refractive lenses and slits. A Dectris Pilatus3 CdTe 2M detector with a pixel size of 172 µm (Vaughan *et al.*, 2020 ▸), placed at a calibrated sample–detector distance of 775 mm, was used to collect the scattered radiation with an exposure time of 30 ms. For each projection angle α about the vertical tomographic *y* axis, the sample was line scanned along *x*. The scanning direction was reversed between consecutive line scans to reduce motor-movement time. A total number of 71 steps in *x* and 61 angular steps of α, with α ε [0°, 180°], were used, giving Δα = 3.0°. The scan procedure was repeated for 29 steps in *y* to measure the full 3D volume, giving a total of 74 × 61 × 29 ≃ 1.6 × 10^5^ recorded diffraction patterns. The total measurement time was ∼2.0 h, including overhead time for motor movements. The diffraction patterns were radially and azimuthally integrated (Ashiotis *et al.*, 2015 ▸) into 2048 radial bins and 64 azimuthal bins to reduce the size of the dataset before further analysis and 3D reconstruction. Prior to the reconstructions, the data were filtered to remove outlier intensities (see Section S11 of the supporting information) and corrected for attenuation. XRD-CT sinograms were generated for each Bragg peak by azimuthally averaging the *q*-integrated scattering after background subtraction. Attenuation-contrast CT parallel-beam sinograms were generated with the Radon transform from the reconstructed tomograms, as the measured attenuation-contrast CT projection data were obtained in the cone-beam geometry [Fig. 1 ▸(*a*)], preventing a direct comparison with the XRD-CT sinograms.

#### 2.2.2. XRDTT reconstruction   

XRDTT reconstruction was carried out using the freely available SASTT software developed by the Coherent X-ray Scattering Group at the Swiss Light Source, Paul Scherrer Institute (Liebi *et al.*, 2015 ▸). The main features will be summarized in the following. The orientational distribution function in each sample voxel **r**′, for a given momentum transfer *q*, was modelled as the absolute square of a spherical harmonic expansion (Liebi *et al.*, 2018 ▸):
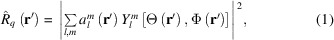
where *a*_*l*_^*m*^(**r**′) are coefficients (here with dimensions of the square root of intensity) for the spherical harmonics 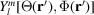 of degree *l* and order *m*. This model is strongly related to pole figures describing anisotropy, and spherical harmonics are routinely used for texture analysis (Roe & Krigbaum, 1964 ▸). For all XRDTT analysis in this article, *m* = 0, *i.e.* the orientation distributions are assumed to be uniaxial. Θ(**r**′) and Φ(**r**′) denote the polar and azimuthal angles in the spherical harmonics coordinate system at position **r**′. The sample coordinates **r**′ are related to the laboratory coordinate system by
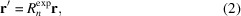
where
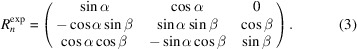
The angles α and β refers to the sample rotation angles used during tomography and are indicated in Fig. 1 ▸(*b*). If only a single tomography axis is used, as for the Pierre shale sample in this work, β = 0. Notably, the rotation matrix 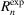 gives the sample *z*′ axis parallel to the laboratory frame *y* axis for α = β = 0°. For each voxel, the parameters *a*_0_^0^(**r**′), *a*_2_^0^(**r**′), *a*_4_^0^(**r**′), *a*_6_^0^(**r**′), θ_op_(**r**′) and ϕ_op_(**r**′) were fitted using a conjugated gradient method with precalculated analytical expressions for the gradients.

From the reconstructed *a*_*l*_^*m*^(**r**′) coefficients, we calculated the uniaxial order parameter, also known as Hermans’ orientation parameter *S*(**r**′) (Hermans *et al.*, 1946 ▸), which gives a measure of the local degree of preferred orientation:
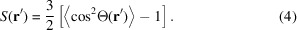
For highly oriented materials, *S* tends towards one. For isotropic materials, *S* is zero.

For the clinochlore 002/kaolinite 002 peaks, the reconstructed spatial variation of the local-crystallite-orientation distribution was obtained using XRDTT. To reduce the amount of streak artefacts in the reconstructions, we applied a filter to remove outlier intensities in the azimuthal intensity distributions deviating more than three absolute deviations from the median. Physically, this filtering approach can be considered as suppressing the larger crystallites to better discern the distributions of the quasi-continuous clay matrix. Other groups have recently demonstrated alternative methods for dealing with outlier intensities in diffraction patterns (Vamvakeros *et al.*, 2021 ▸).

### 2.3. Attenuation-contrast CT   

Attenuation-contrast µCT of the Pierre shale sample was performed with a home-laboratory cone-beam CT instrument of type Nikon XTH 225, equipped with a PerkinElmer (Waltham, USA) 1620 CN CS detector with 2000 × 2000 pixels. A tungsten reflection target was used with an acceleration voltage of 180 kV. No beam-conditioning filter was used, and 1001 equally angularly spaced projections were obtained with α ε [0°, 360°]. The 3D attenuation-coefficient map was reconstructed using the Nikon software *X-TEK CT Pro 3D*, based on the Feldkamp–Davis–Kress algorithm (Feldkamp *et al.*, 1984 ▸), giving an isotropic voxel size of 5.0 µm.

## 3. Results and discussion   

### 3.1. The mineral composition and clay mineral orientation in Pierre shale   

**Figure 2:**
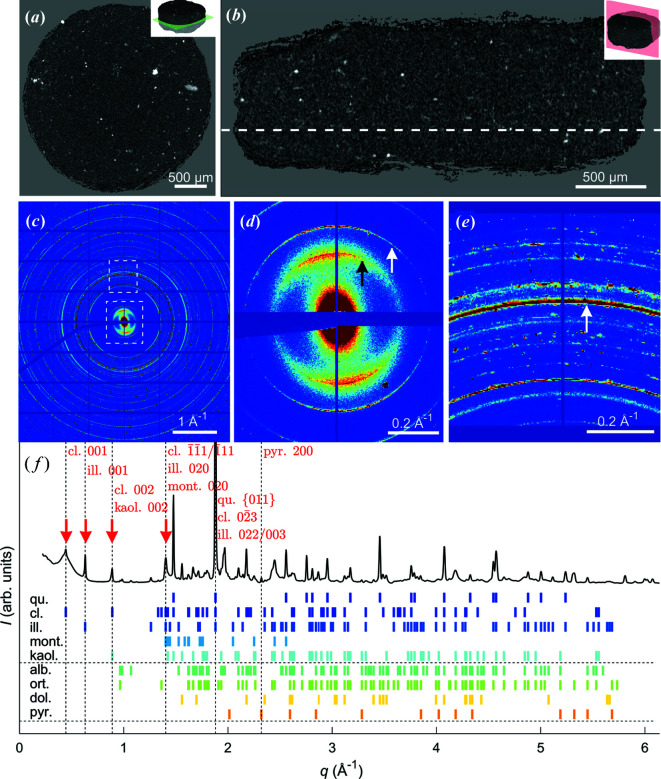
An overview of the Pierre shale sample. (*a*), (*b*) Orthogonal attenuation-contrast CT cross sections. (*c*) A single diffraction pattern obtained from the sample centre with insets shown in (*d*) and (*e*). There is strong texture in the clinochlore 001 and illite 001 peaks, marked with black and white arrows, respectively, in (*d*). The white arrow in (*e*) points to the overlapping Bragg peaks of quartz {011}, clinochlore 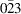 and illite 022/003. The grid and the missing data are due to detector panel gaps and the beam stop. (*f*) An indexed diffractogram revealing the sample composition. The low-*q* Bragg peaks marked with red arrows all display pronounced texture. The intense overlapping peaks of quartz {011}, clinochlore 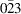 and illite 022/003 have been cut in (*f*) for display purposes. Abbreviations: qu., quartz; cl., clinochlore; ill., illite; mont., montmorillonite; kaol., kaolinite; alb., albite; ort., orthoclase; dol., dolomite; and pyr., pyrite.

Home-laboratory attenuation-contrast µCT of the shale sample is presented in Figs. 2 ▸(*a*) and 2 ▸(*b*). The attenuation was approximately uniform across the whole sample, except for some highly attenuating regions appearing as bright spots in the reconstructed tomograms. Owing to unresolved nanoscale grains and inclusions in the heterogeneous sample, giving strong partial volume effects, these CT data cannot be segmented in a reproducible and meaningful manner. This statement is emphasized by the high-resolution holography cross section presented in Section S3, clearly demonstrating that the typical grain size is much smaller than the chosen XRD-CT voxel size. While it is tempting to claim that the strongly absorbing regions could be electron-rich iron-containing inclusions of pyrite, it is, as we shall demonstrate, perilous to do so without further analysis.

**Figure 3:**
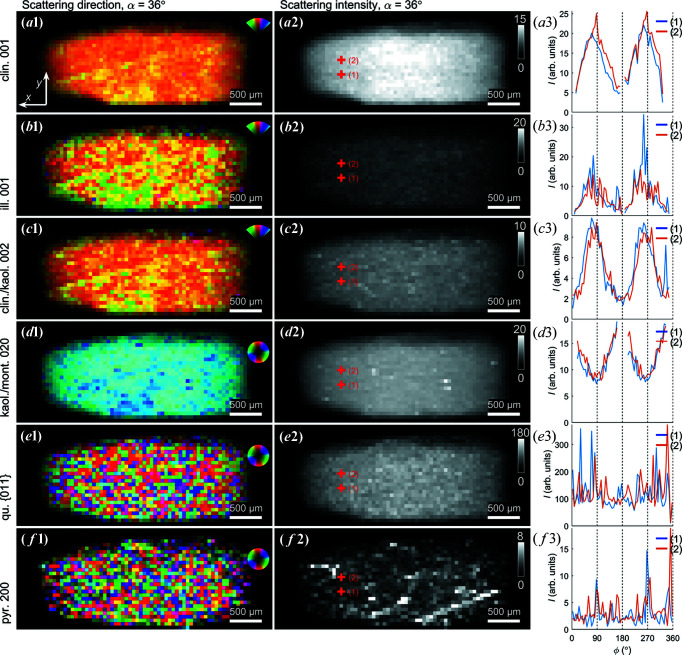
Orientation, intensity and orientation distribution of selected Bragg diffraction peaks, observed for the projection angle α = 36°. Note the band-like orientation feature stretching diagonally across the sample. (*a*1) Dominant scattering direction for the clinochlore 001 Bragg peak, colour coded as shown in the inset. (*a*2) Azimuthally integrated intensity of the clinochlore Bragg peak. (*a*3) Azimuthal intensity variations for two points indicated in (*a*2). (*b*)–(*f*) Similarly for the illite 001, clinochlore 002/kaolinite 002, kaolinite 020/montmorillonite 020, quartz {011} (overlapping with clinochlore and illite) and pyrite {200} Bragg peaks, respectively.

The measured XRD-CT diffraction patterns [Figs. 2 ▸(*c*)–2 ▸(*f*)] consist of (i) quasi-continuous Debye–Scherrer rings originating from the many randomly oriented small-grained polycrystalline minerals, and (ii) numerous interspersed high-intensity Bragg peaks corresponding to larger and/or better-oriented crystallites within the scattering volume (Wilchinsky, 1951 ▸). By averaging all the measured diffraction patterns (∼10^5^) for the sample, a volume-averaged ‘master’ diffractogram was obtained, which we in a related study (Chattopadhyay *et al.*, 2020 ▸) have shown by Rietveld refinement to be consistent with the presence of quartz and illite, with smaller amounts of montmorillonite, kaolinite, clinochlore, albite, orthoclase and pyrite. Indexed peaks based on the volume-averaged diffractogram are shown in Fig. 2 ▸(*f*). A total of 141 Bragg peaks in the measured range *q* = 0.22–6.1 Å^−1^ were identified and compared with tabulated values from the American Mineralogist Crystal Structure Database (http://rruff.geo.arizona.edu/AMS/amcsd.php). Notably, as there were numerous Bragg peaks present giving substantial peak overlap, only clinochlore 001, illite 001, clinochlore 002/kaolinite 002, kaolinite 020/montmorillonite 020, quartz {011} (overlapping with clinochlore and illite) and pyrite {200} were used for further analysis, see Fig. 3 ▸.

An example of a single-exposure diffraction pattern obtained with the pencil beam penetrating the sample near its centre is shown in Figs. 2 ▸(*c*)–2 ▸(*e*), where many sharp Debye–Scherrer rings can be observed. Most of the rings are essentially isotropic, with the important exception of the low-*q* rings originating from the clay phases. The broad clinochlore 001 and illite 001 Bragg peaks exhibit strong texture with dominant scattering in the same direction, understood to originate from the clay crystallites being aligned with the bedding planes (Sander, 1934 ▸; Wenk *et al.*, 2010 ▸).

As a first step towards quantifying the anisotropic intensity distribution of the clay minerals we used Fourier analysis to retrieve the integrated intensity and dominant scattering direction (Bunk *et al.*, 2009 ▸), with selected results presented in Fig. 3 ▸. The maps shown are all from the projection with α = 36°, yet for different narrow *q* regions, thus targeting different minerals in the sample. This analysis was carried out without tomographic reconstruction, only quantifying the azimuthal intensity variations (texture) present in the recorded diffraction patterns. The background scattering was subtracted, with the background estimated from averaging the intensity at the peak shoulders, and the intensity was averaged radially (along *q*) across each Bragg peak. The scattering patterns from the broad clinochlore 001, illite 001, clinochlore 002/kaolinite 002 and kaolinite 020/montmorillonite 020 peaks were found to be highly directional in all sample regions [Figs. 3 ▸(*a*)–3 ▸(*d*)], whereas the scattering from pyrite {200} and quartz {011} was isotropic [Figs. 3 ▸(*e*) and 3 ▸(*f*)]. The azimuthal intensity variations from selected points are shown in the third column. Consistently, the preferred orientation of clinochlore 001, illite 001 and clinochlore 002/kaolinite 002 is seen to be orthogonal to kaolinite 020/montmorillonite 020 [*cf*. Figs. 3 ▸(*a*1)–3 ▸(*c*1) versus 3 ▸(*d*1)]. The integrated scattering intensity suggests that the clay minerals and quartz were distributed rather evenly across the whole sample volume, as expected from the uniform appearance of the sample, whereas localized regions of pyrite were found. Still, from the spiked azimuthal intensity variations at selected points [*e.g.* Fig. 3 ▸(*b*3)], it is evident that there were also contributions from larger clay crystallites (see below).

Uniquely for the presented α = 36° projection, a band of variation in the clay signals can be discerned across the sample. This feature stretching diagonally across the sample was observed for the broad peak containing clinochlore 001, illite 001, and clinochlore 002/kaolinite 002 in both preferred orientation and integrated intensity. These slight variations in the scattered intensity could originate from density gradients or be due to large favourably oriented crystallites. The fact that these bands could only be seen for one projection angle suggests that the observed band is in fact a plane across the sample that has a well defined angle with the rotation axis, thus being blurred for all the other projection angles. While tensorially reconstructing the plane proved elusive, the observed plane might be associated with shale fissility.

The clay diffraction signals varied smoothly across the projections with well defined preferred orientation, however the signals from quartz [(Fig. 3 ▸(*e*)] and pyrite [Fig. 3 ▸(*f*)] were qualitatively different. Note that α-quartz (SiO_2_) is trigonal (space group *P*3_2_21) and pyrite (FeS_2_) is simple cubic (space group 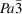). The many and strong scattering contributions are interpreted to be caused by a combination of heavier elements, larger crystallites, and the Bragg scattering condition being better fulfilled. With the resolution of our experiment, the quartz particles appear to be present across the full sample volume and randomly oriented with abrupt changes in orientation between adjacent exposures, strongly suggesting that these mineral grains are disconnected and of sub-voxel size. The quartz {011} reflections by symmetry have a multiplicity of 12, which must be accounted for if we desire a more quantitative interpretation of the signal. Similar considerations apply to the pyrite {200} signal (multiplicity of six), with randomly oriented grains found across the whole sample. Pyrite tended to be localized to certain regions [Fig. 3 ▸(*f*)] and the intensity distribution suggests a density correlation over longer distances.

### 3.2. Retrieving the clay mineral orientation by XRDTT   

Numerical XRDTT reconstructions, as described in Section 2.2.1, allowed determining the local orientation of the clay crystallites from the measured textured scattering. For XRDTT, multiple tomography axes have previously been used to provide sufficient sampling of the reciprocal space (Liebi *et al.*, 2015 ▸, 2018 ▸; Schaff *et al.*, 2015 ▸). However, it remains debated how much redundancy is needed for faithfully reconstructing preferred orientation distributions in a given sample, a topic that was already raised by Liebi *et al.* (2018 ▸). Intuitively, it appears that for ‘well behaved’ samples with (i) a global uniaxial orientation and (ii) a broad orientational distribution function associated with each voxel, a single tomographic rotation axis co-linear with the unique axis of the sample is sufficient to reconstruct maps of crystallite preferred orientation. We provide support for this claim using a piglet bone/cartilage sample, measured with multiple tomography axes, by comparing reconstructions based on the full dataset with a single-axis subset, see Section S7. In essence, when the beam energy is high (*E* = 50.00 keV), the Ewald sphere is nearly flat at the Bragg peaks studied here; for clay, *q* ≃ 0.6–1.4 Å^−1^ and 2θ ≃ 1.4–3.2°. The Bragg angle θ is thus an order of magnitude smaller than the observed width of the assumedly uniaxial (*m* = 0) orientation distributions. Thus, when scanning the sample 180° around the tomography axis, the uniaxial orientation distributions (‘pole figures’) of all the low-*q* diffraction peaks will be measured to good accuracy.

**Figure 4:**
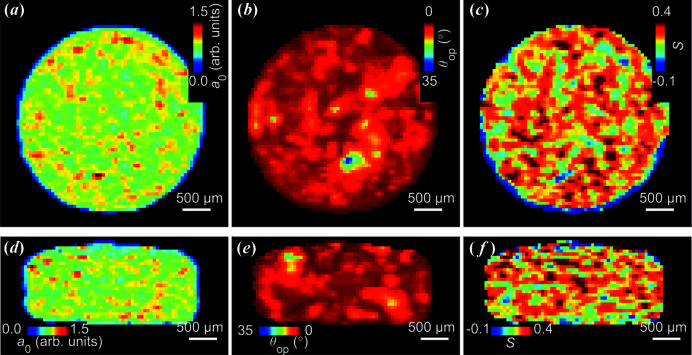
Reconstructed XRDTT orthogonal cross sections of the overlapping clinochlore 002/kaolinite 002 Bragg peaks. (*a*), (*d*) Isotropic scattering *a*_0_. (*b*), (*e*) Directionality of scattering relative to the tomography axis. (*c*), (*f*) The reconstructed Hermans’ parameter *S*, *cf.* equation (4).

Reconstructed XRDTT cross sections of the clay sample are shown in Fig. 4 ▸, exactly matching the µCT cross sections in Fig. 2 ▸. In Fig. 4 ▸, cross-sectional maps of the overlapping clinochlore 002/kaolinite 002 peak are displayed showing (i) the local isotropic scattering intensity *a*_0_(**r**′), (ii) the uniaxial (polar) angle θ_op_(**r**′) of the preferred orientation axis with respect to the *z*′ axis [Fig. 1 ▸(*c*)], and (iii) the Hermans’ parameter *S*(**r**′) describing the degree of orientation. As seen in the reconstructed *a*_0_(**r**′), the density of clay minerals was approximately uniform, see Figs. 4 ▸(*a*) and 4 ▸(*d*). Despite local variations, the clay mineral orientation was found to be directed mainly in one direction, as expected for a relatively small shale sample [Figs. 4 ▸(*b*) and 4 ▸(*e*)]; however the degree of preferred orientation, described by the Hermans’ *S* parameter, varies [Figs. 4 ▸(*c*) and 4 ▸(*f*)]. Similar XRDTT reconstructions were made for the broad low-*q* peak which includes clinochlore 001; however the reconstructed preferred orientation directions differed from the clinochlore 002 case (Fig. 4 ▸), presumably due to the large amount of Bragg peak overlap, see Section S8. The mean preferred orientation direction of the [001] clay axis was found to be given by the angles ϕ_op, mean_ = 80.1° and θ_op, mean_ = 6.4° by averaging the scattering directions for all voxels in the sample. The reconstructions were robust; different initial values for the XRDTT reconstructions provided similar results. Choosing the sample *z*′ axis to (approximately) coincide with the predominant clay mineral orientation, as governed by equation (3), appears advantageous for numerical reconstruction, as the resulting θ_op_(**r**′) is close to zero, and then θ_op_(**r**′) and ϕ_op_(**r**′) are far from gimbal lock.

**Figure 5:**
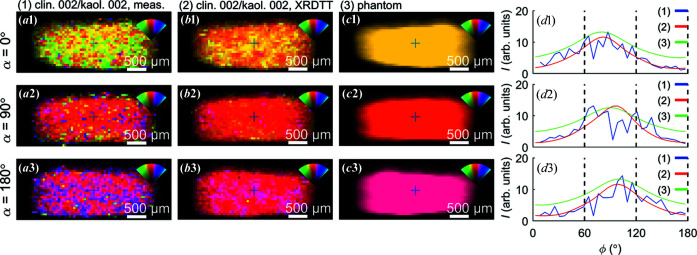
Comparison of the measured directional clay scattering with forward projections of the XRDTT reconstruction and the simple phantom. (*a*) Measured scattering of clinochlore 002 and kaolinite 002 for 0, 90 and 180°. (*b*) Forward-projected scattering from XRDTT reconstruction. (*c*) Forward-projected scattering from the phantom, where all voxels have been assigned the same direction. (*d*) Azimuthal intensity distributions for points marked with (+).

As a plausibility test of the XRDTT results, the forward-simulated scattering from the reconstructed model was calculated and found to be similar to the measured scattering, as shown in Fig. 5 ▸. Additionally, to support the finding of the dominant clay orientations in the sample, we simulated the forward scattering from a numerical phantom of the same size and shape as the measured sample, but with all voxels assigned the same orientation. Setting ϕ_op_ = 90.0° and θ_op_ = 10.0° reproduced the gross features of the measured projection data, *cf*. Fig. 5 ▸. By comparing the α = 0° and α = 180° projections, the dominant scattering directions are seen to be symmetrically flipped about the vertical axis for clinochlore 001 (coinciding with illite 001). This observation implies that the reciprocal lattice vector for clinochlore 002/kaolinite 002 was oriented approximately perpendicular to the incoming beam direction for these projections, and thus directly gives an estimate of the tilt of the bedding-plane normal with respect to the tomography axis.

### 3.3. Identifying clastic inclusions by combining XRD-CT and attenuation-contrast CT   

**Figure 6:**
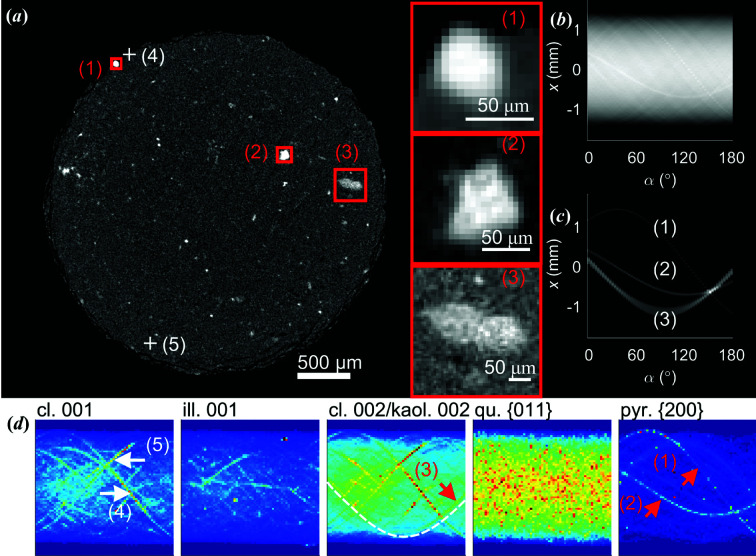
Identification of clastic mineral composition by comparing attenuation- and XRD-contrast sinograms. (*a*) An attenuation-contrast cross section of the sample. Three inclusions are marked with red rectangles and have been magnified. A sinogram (Radon transform) of the entire cross section in (*a*). (*c*) A sinogram made by including only the bright regions indicated by the red rectangles in (*a*). (*d*) XRD-CT sinograms based on clinochlore 001, illite 001, clinochlore 002/kaolinite 002, quartz {011} (overlapping with clinochlore and illite), and pyrite {200}. All sinograms are displayed with different intensity scales. The white dashed line is a guide to the eye.

When we introduced the attenuation-contrast CT cross section in Fig. 2 ▸(*a*), we emphasized the fundamental ambiguities associated with the mineral identification and segmentation. We shall now demonstrate how this problem can be resolved by combining the two datasets. Fig. 6 ▸(*a*) shows the same attenuation-contrast CT cross section as in Fig. 2 ▸(*a*), with three strongly attenuating inclusions highlighted. Additional cross-sectional views of these three inclusions are provided in Section S2. In the XRD-CT sinograms, several discontinuous traces are present, see Fig. 6 ▸(*d*) and Section S10. These discontinuities are caused by the crystallites rotating in and out of the diffraction condition, and imply that tomograms cannot be straightforwardly reconstructed by adapting methods from conventional CT. Attempting to use *e.g.* FBP (after filtering and averaging) gave substantial streak artefacts, precluding reliable reconstruction and interpretation, see also Figs. S11.1 and S11.2 of the supporting information.

Instead of automated XRDTT analysis, we thus relied on studying the spatial distributions of the minerals in the sample by direct comparison of the XRD-CT sinograms with attenuation-contrast CT sinograms. From the close correspondence between the co-registered sinograms we were able to map specific minerals in the sample, without being affected by streak artefacts in the tomograms. Sinograms derived from diffraction patterns of clinochlore 001, illite 001, clinochlore 002/kaolinite 002 (overlapping with clinochlore and illite), and pyrite {200} are shown in Fig. 6 ▸(*d*). For pyrite {200}, the sinogram curves corresponding to particles marked (1) and (2) have approximately constant intensity for all projection angles α, implying that for every rotation step of the sample, approximately the same number of crystallites must satisfy the Bragg condition. By studying the raw diffraction patterns, we could indeed confirm that these pyrite particles consist of several randomly oriented crystalline grains, see Figs. S4.3 and S4.4. The full diffractograms for the regions (1) and (2) could be reconstructed as shown in Fig. S12, consistently showing presence of the pyrite Bragg peaks. Region (3) in Fig. 6 ▸ matched curves in the clinochlore 002/kaolinite 002 sinograms [Fig. 6 ▸(*d*)], firmly rejecting the idea that these strongly attenuating features are all electron-rich minerals. There were features, exemplified by (4) and (5) in Fig. 6 ▸(*a*), with a strong XRD-CT signal corresponding to clinochlore, that did not give a distinct signal in the attenuation-contrast CT. In addition to the comparison between the attenuation-contrast CT and XRD-CT sinograms made to identify the mineralogy in the selected regions in Fig. 6 ▸(*a*), the correlation between the attenuation-contrast CT sinograms and the XRD-CT sinograms was calculated, see Section S13. This procedure gave similar information as XRD-CT reconstruction (see Sections S11 and S12), however avoiding streak artefacts originating from the CT reconstruction, and is thus suggested as an alternative method for mineral localization in under-sampled measurements.

Other strategies exist to retrieve the spatial and orientational distributions of crystallites that are large compared with the voxel size. Notably, 3DXRD (Poulsen, 2004 ▸) allows accurate crystalline mapping, also including orientation, provided that diffraction spots originating from individual grains can be uniquely separated, and the diffraction signal predominantly originates from larger grains, contrary to the case observed in the present work.

Our motivation for this work has been to study the spatial distributions and orientation of minerals in shales to better understand their physicochemical properties. We have investigated if we could obtain the clay mineral orientation by applying XRDTT, similar to what has previously been carried out on bone by us (Mürer *et al.*, 2021 ▸) and others (Liebi *et al.*, 2015 ▸, 2018 ▸; Grünewald *et al.*, 2020 ▸); however, here we used a single tomography axis, thus significantly reducing the experimental complexity and the reconstruction computing time. In tensor tomography, when the solution is found by optimization of a large amount (∼10^6^) of parameters, the use of multiple tomography axes can be beneficial in adding more sampling points to prevent the optimization from stagnating in local minima. Similar orientation patterns (Fig. 4 ▸) were found with different initial conditions of the angular optimization, supporting both the validity of the orientational maps and the experimental procedure. We refer again to the study of the bone and cartilage, reported in Section S7, for a detailed comparison of reconstructions with one or two tomographic axes.

Shales contain pores and mineral grains on length scales from nanometres to centimetres (Ma *et al.*, 2017 ▸), and in this experiment we were limited by (i) the 50 µm voxel size, defined by the size of the beam in XRD-CT, and (ii) the limited resolution in *q* due to the placement of the detector far from the sample. We have previously demonstrated that even small fragments (∼4 µm diameter) of Pierre shale can consist of several mineral phases (Chattopadhyay *et al.*, 2020 ▸). However, the large field of view used in this experiment enabled us to non-destructively map out larger (∼50–100 µm) mineral features within a millimetre-sized volume, a feat that to our knowledge would have been impossible with other chemistry-sensitive techniques used to characterize shale, such as electron backscatter diffraction (Prior *et al.*, 1999 ▸). XRD-CT and XRDTT are clearly promising pathways towards a much richer understanding of the micro- and nano-scale features in shales, deserving of the efforts of implementing a fully automated systematic analysis.

## 4. Conclusions   

We have utilized XRDTT to study scattering from clay minerals and high-density inclusions in Pierre shale. Orientation maps of the clay minerals have been obtained, demonstrating that texture information from clay minerals can be extracted in XRDTT by using a single tomography axis as in conventional CT. The abundant clay minerals in shales were demonstrated to exhibit strong global preferred orientation as expected, and with XRDTT also minor variations could be mapped in 3D. We have demonstrated that a systematic use of sinograms based on combining the attenuation-contrast and diffraction-contrast data allows identification of the mineralogy of selected regions. Inclusions were identified to contain multi-grained pyrite or clinochlore crystallites.

## 5. Related literature   

The following references are cited in the supporting information for this article: van Aarle *et al.* (2016 ▸), Hughes *et al.* (1989 ▸), Kak & Slaney (1987 ▸), Meneghini *et al.* (2003 ▸), Vamvakeros *et al.* (2015 ▸), Wenk & Heidelbach (1999 ▸).

## Supplementary Material

Supporting information. DOI: 10.1107/S205225252100587X/ro5025sup1.pdf
