# Supplementary material for: Orientational mapping of minerals in Pierre shale using X-ray diffraction tensor tomography
Source: IUCrJ. 2021 Jul 17;8(Pt 5):747–56. doi: 10.1107/S205225252100587X (PMC8420771; doi:10.1107/S205225252100587X)
Supplement: Supplementary file 1 [file m-08-00747-sup1.pdf]

IUCrJ

Volume 8 (2021)

Supporting information for article:

Orientational mapping of minerals in Pierre shale using X-ray diffraction tensor tomography

Fredrik K. Mürer, Aldritt Scaria Madathiparambil, Kim Robert Tekseth, Marco Di Michiel, Pierre Cerasi, Basab Chattopadhyay and Dag W. Breiby

S1. Pierre shale

Figure S1 shows pieces of Pierre shale and the studied sample mounted onto a polymer pin which was attached to the goniometer head. The sample was cut to the shape of a cylinder, with approximate diameter 3.5 mm and height 1.3 mm.

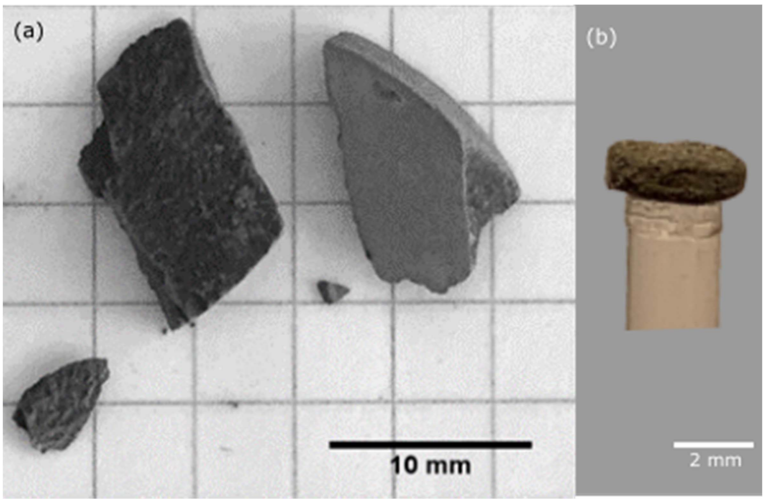

Figure S1 Sample preparation. (a) Pierre shale outcrop. (b) Photograph of the shale sample mounted onto a pin, which was attached to the goniometer head.

S2. Attenuation-contrast cross-sections

Orthogonal views are provided of the high-density inclusions, shown in Figs. 2 and 6 in the main article, see Fig S2.

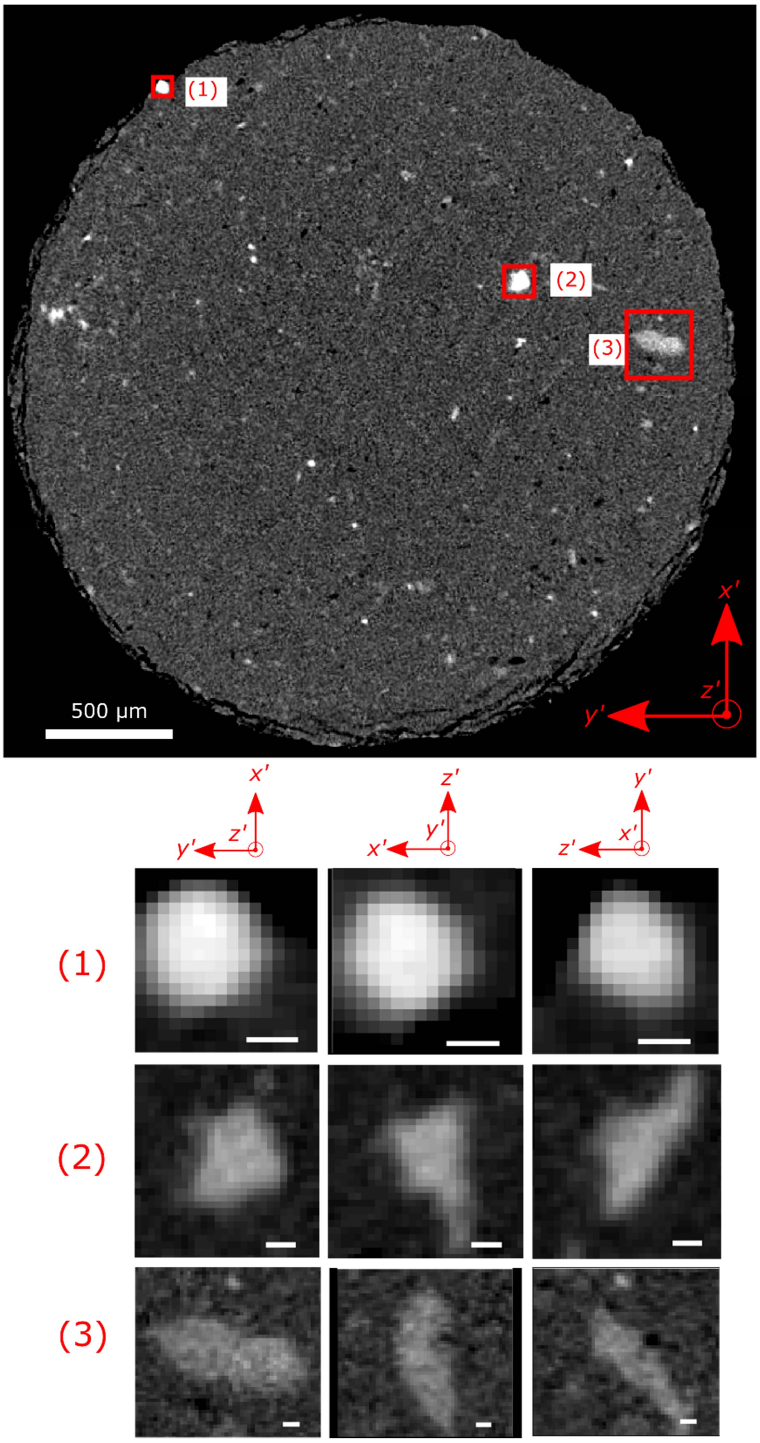

Figure S2 Attenuation-contrast CT cross-sections. a) The cross-section displayed in the article. b-j) Orthogonal cross-sections of the high-density particles marked with rectangles. The scalebars in the insets are 25 µm. Different intensity scaling has been used for the cross-section in (a) and the various insets to enhance the contrast.

S3. High-resolution synchrotron phase-contrast imaging of Pierre shale

To study the local variations in grain sizes in the sample we did phase-contrast local tomography at the beamline ID16 at the ESRF, Grenoble. A cross-section obtained from the exact same Pierre shale sample as measured with XRD-CT and presented in the main article is shown in Fig. S3. The reconstructed voxel size is 60 nm, allowing identification of single grains, however the field-of-view with this method is limited to only cover a small region within the sample centre.

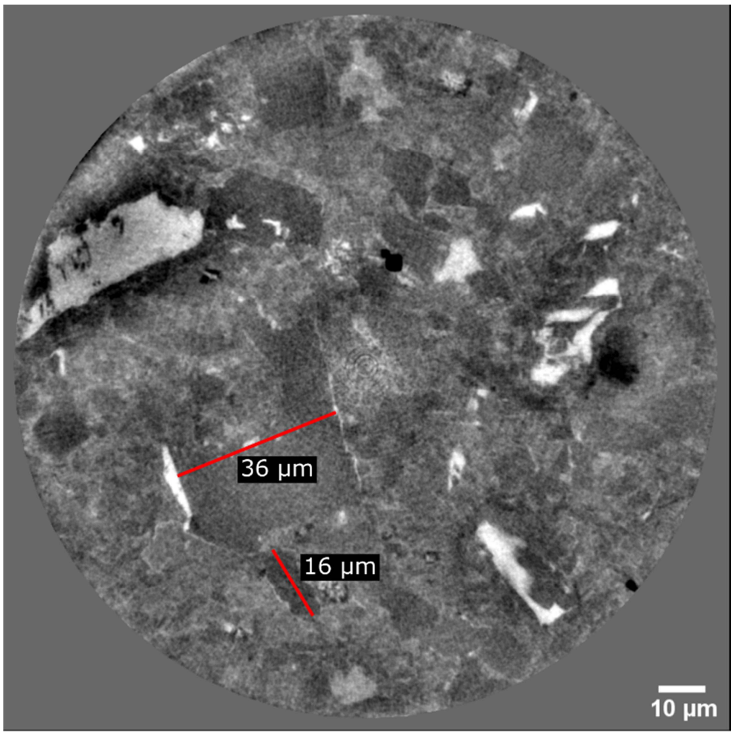

Figure S3 Cross-section of Pierre shale, imaged with high-resolution synchrotron phase-contrast CT. Local tomography propagation phase-contrast CT, measured at ID16, ESRF with a reconstructed voxel size of 60 nm. The exact same sample as measured in the manuscript, but with a smaller field of view. The approximate sizes of larger grains have been indicated.

S4. Oriented scattering from minerals

Figures S4.1 and S4.2 show the directional scattering for the shale sample from 6 different Bragg peaks with the sample orientation for $\alpha = 0°$ and $\alpha = 180°$, respectively, complementary to Fig. 3 in the main article where $\alpha = 36°$ is shown. Each pixel (x,y) corresponds to a single measured diffraction pattern.

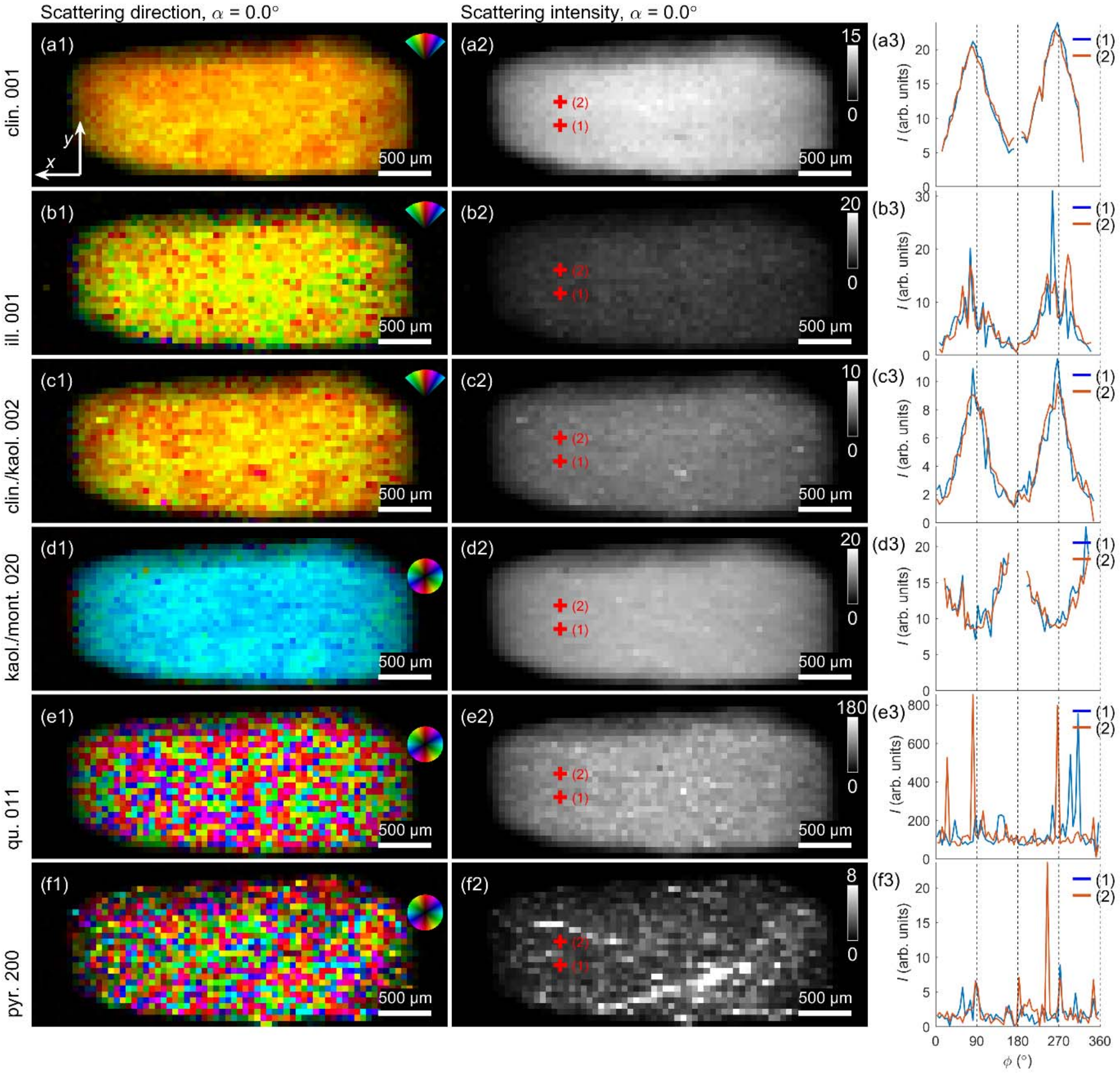

Figure S4.1 Dominant scattering direction and integrated scattered intensity obtained for a single projection with $\alpha = 0°$.

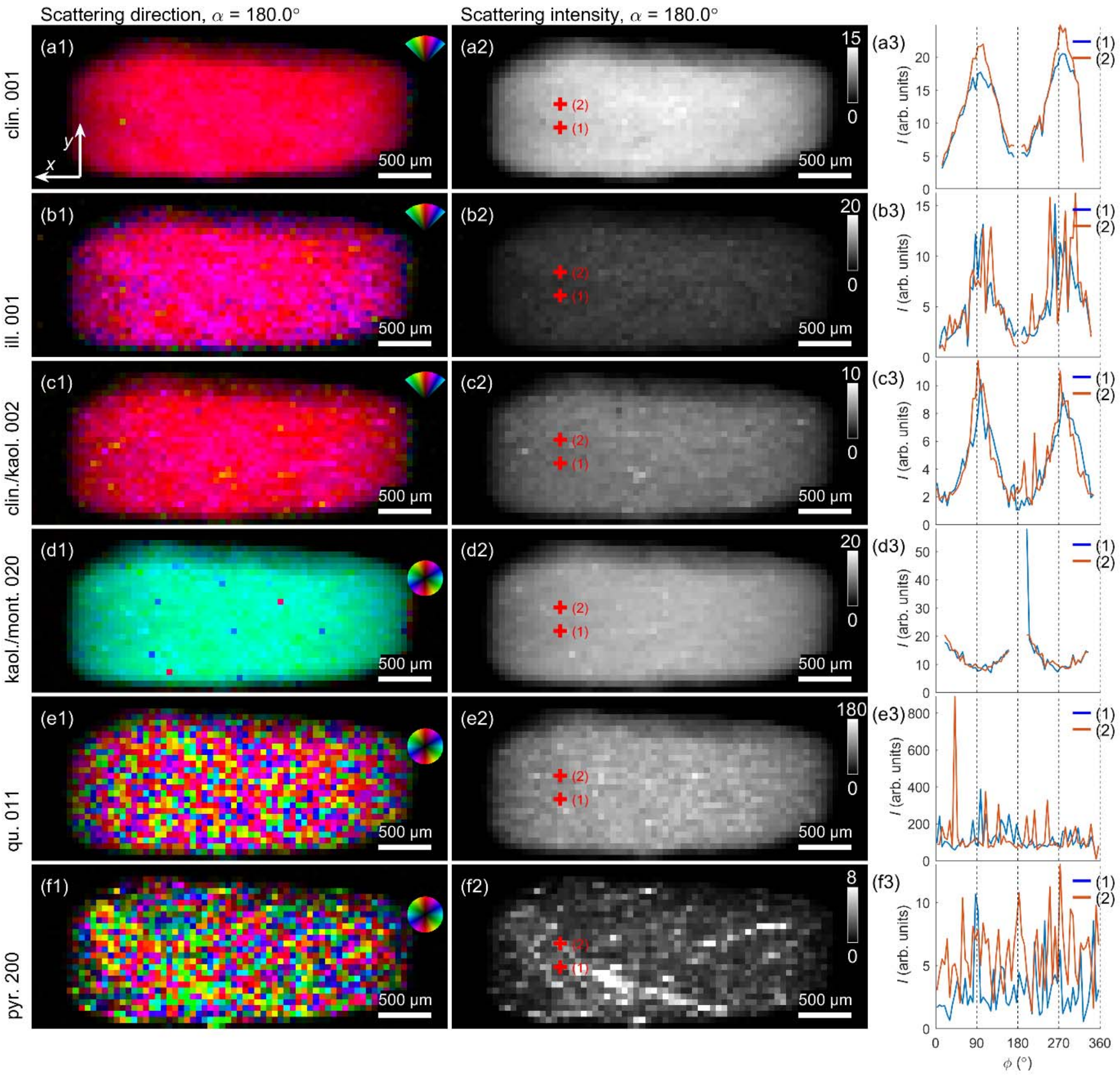

Figure S4.2 Dominant scattering direction and integrated scattered intensity obtained for a single projection with α = 180º.

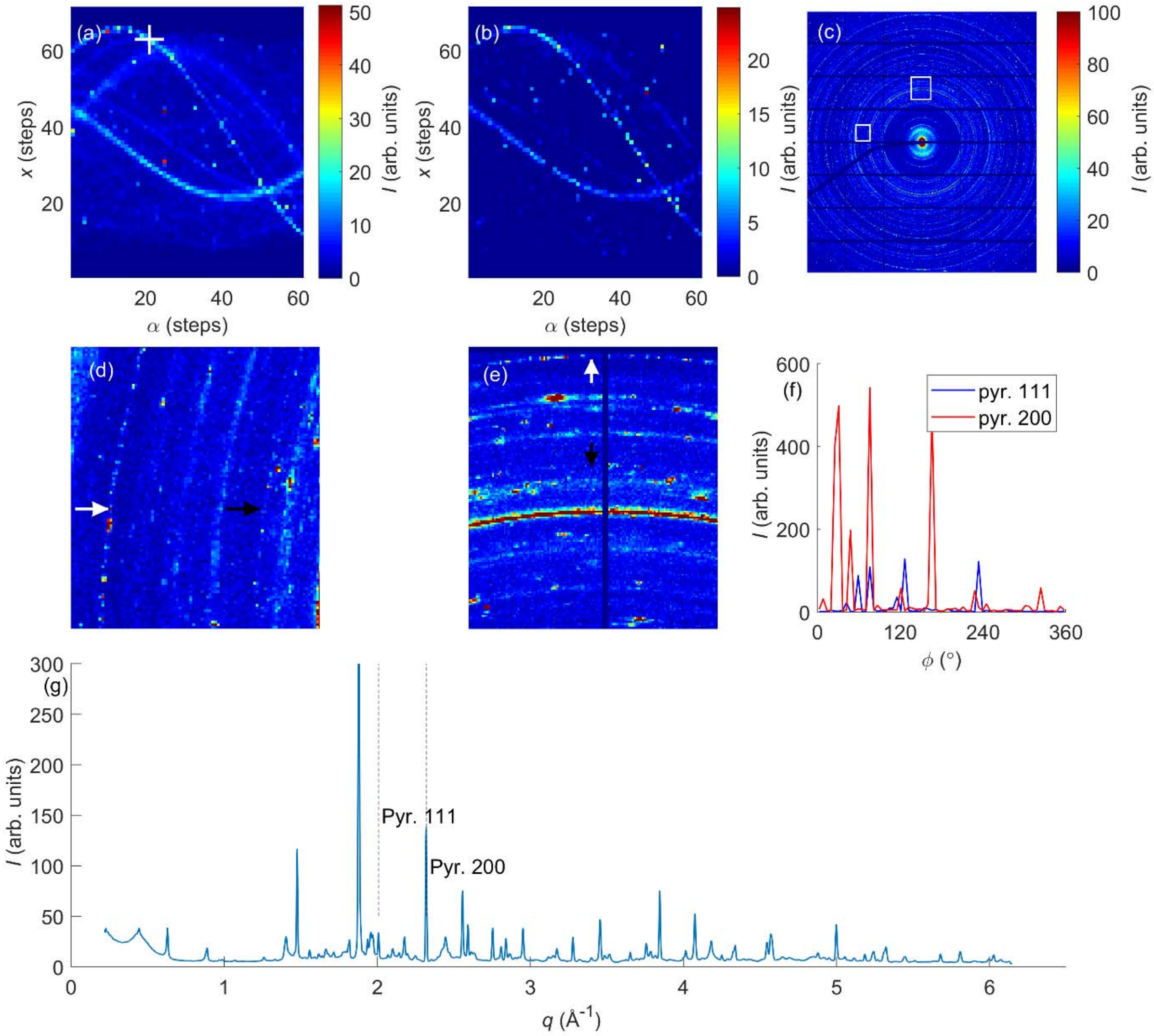

Figure S4.3 Scattering from the pyrite-containing region marked (1) in Figure 6a. (a) Sinogram of pyrite {200}. (b) Unfiltered sinogram of pyrite {111}. (c) Diffraction pattern corresponding to the white (+) in (a). (d-e) Magnified regions from (c). The black and white arrows point to the pyrite {111} and {200} Bragg peaks, respectively. (f) Azimuthal intensity variation of pyrite {111} and {200}. (g) Azimuthal integrated intensity of the diffraction pattern in (c). Steps in x and α are 50 µm and 3.0°, respectively.

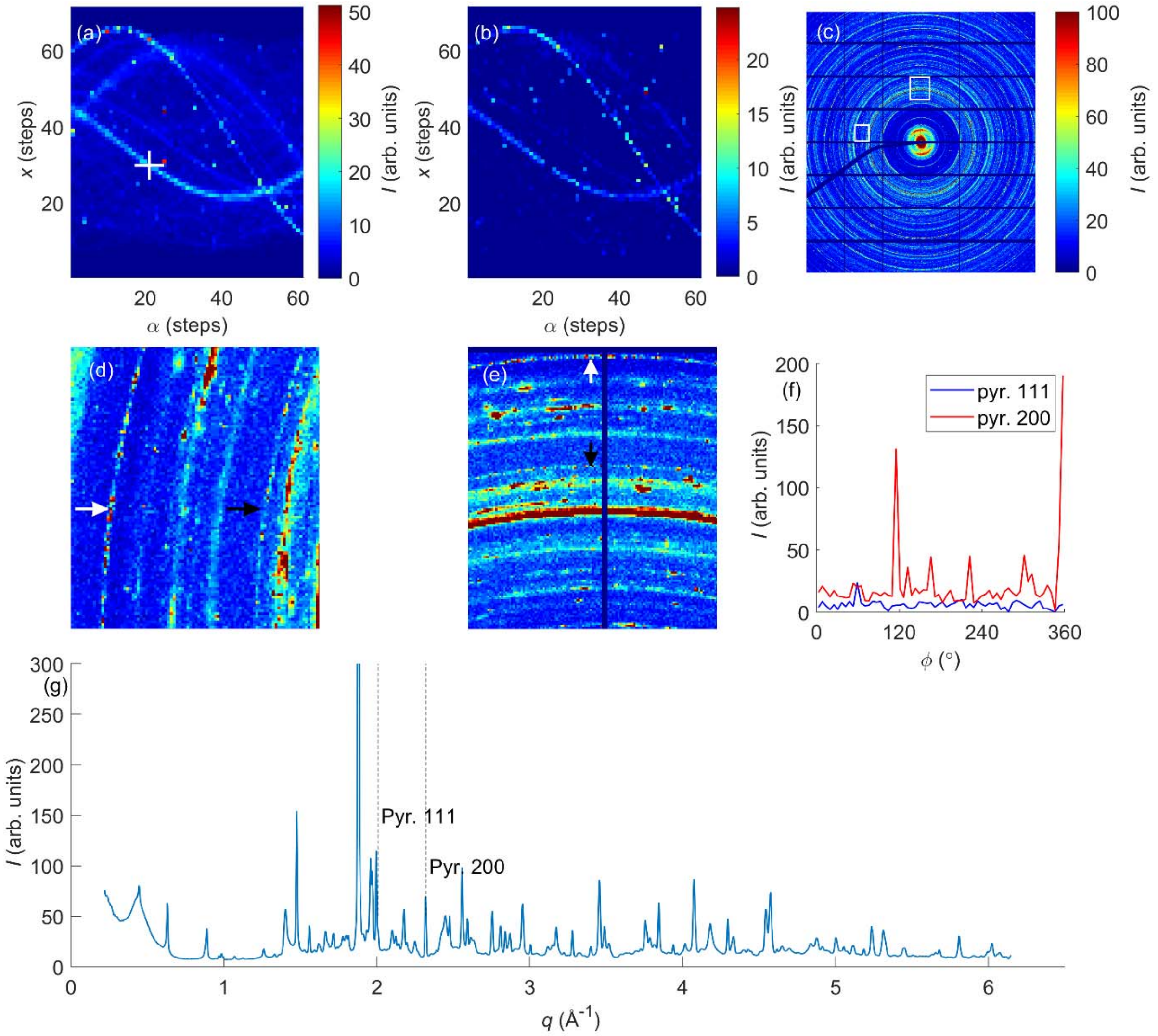

Figure S4.4 Scattering from the pyrite-containing region marked (2) in Figure 6a. (a) Sinogram of pyrite {200}. (b) Unfiltered sinogram of pyrite {111}. (c) Diffraction pattern corresponding to the white (+) in (a). (d-e) Magnified regions from (c). The black and white arrows point to the pyrite {111} and {200} Bragg peaks, respectively. (f) Azimuthal intensity variation of pyrite {111} and {200}. (g) Azimuthal integrated intensity of the diffraction pattern in (c). Steps in x and α are 50 µm and 3.0°, respectively.

S5. Sample attenuation corrections

Beam attenuation artefacts were observed in the reconstructed tomograms in both the isotropic XRD-CT reconstruction and in the orientational XRDTT reconstruction. As no attenuation measurements were done during the experiment, we devised a simple attenuation correction based on the reconstructed cross-sections. The attenuation correction was applied for the reconstruction of all sinograms, except where the diffraction patterns had been filtered before integration. An uncorrected reconstructed cross-section of the illite 001 scattering is shown in Figure S5a. From the line plot in Figure S5c, it is seen that for the uncorrected tomogram (having the attenuation coefficient $\mu = 0$), lower intensities occur close to the sample centre, where the beam is most attenuated upon traversing the sample.

The sample attenuation correction of the sample was done by:

(i) Performing an initial reconstruction to make a sample mask.
(ii) Assigning an effective attenuation coefficient μ, constant for the whole sample volume.
(iii) Forward projection of the simulated transmission sinogram.
(iv) Correcting the sinogram by the simulated transmission sinogram.

The attenuation correction was applied for both isotropic XRD-CT and for XRDTT. Examples are shown in Figure 4.2. The absorption correction in isotropic XRD-CT was applied separately for each q. An attenuation coefficient of $\mu = 0.005$ vox^{-1} was found to mitigate the absorption artefacts, cf. Fig. S5(b) and (c).

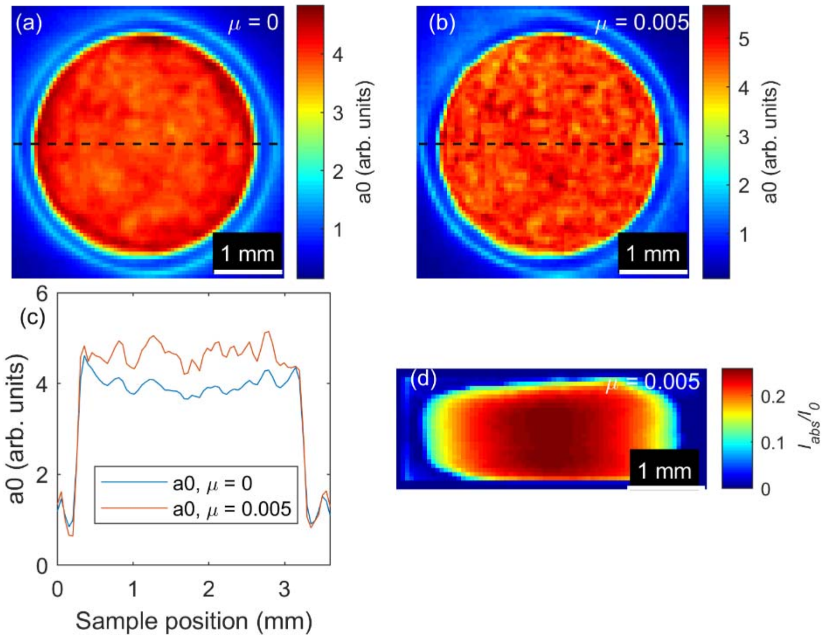

Figure S5 Absorption corrections for the Pierre shale sample. (a) Reconstructed isotropic tomogram of illite 001 using no absorption correction. Higher intensity regions can be observed close to the sample edges. (b) Reconstructed tomogram of illite using an absorption correction of $\mu = 0.005$ vox^{-1}. c) Line plot through cross sections in (a) and (b). Higher constructed intensities can be observed close to the sample edges in the uncorrected reconstruction. (d). Forward simulated azimuthally integrated intensity projection ($\alpha = 0°$), using an absorption coefficient of $\mu = 0.005$ vox^{-1}. The maximum absorption in the projection is estimated to be approximately 20%.

S6. XRDTT reconstruction

XRDTT reconstruction was done by following the approach described by (Liebi *et al.*, 2018) and by using the small-angle scattering tensor tomography (SASTT) package developed by the coherent X-Ray Scattering Group at the Paul Scherrer Institute, Villigen, Switzerland. An additional rotation matrix was used to align the sample z'-axis with the laboratory y-axis, see Fig. 1 and Eqs. 2 and 3. Parameters used for XRDTT reconstruction of the clinochlore 001 and clinochlore 002 peaks are found in Table S4. The parameter names refer to entries in the SASTT MATLAB package.

Table S6 Initial parameters used for XRDTT reconstruction of clinochlore 001 and clinochlore 002 Bragg peaks. Cf. (Liebi *et al.*, 2018) for a full description.

**STEP**	**Parameter**	**Clinochlore 002/kaolinite 002**
Symmetric intensity	regularization	not applicable
	regularization_angle	not applicable
	itmax	20
SH angles	regularization	not applicable
	regularization_angle	not applicable
	itmax	50
	a1_init	1
	a2_init	-20
	a3_init	10000
	l	[0 2 4 6]
	m	[0 0 0 0]
	theta_init	pi/4
	phi_init	pi/4
SH coefficients	regularization	1
	regularization_angle	not applicable
	kernel3D	window3(3,3,3,@hamming)
	itmax	20
	l	[0 2 4 6]
	m	[0 0 0 0]
	a	[0.001 -0.0001 0.001 0.0001]
Combination of all parameters	itmax	50
	regularization	not applicable
	regularization_angle	not applicable
	regularization_angle_coeff	2.00

S7. XRDTT of hydroxyapatite crystallites in bone using a single tomography axis

To further strengthen our assumption of using a single tomography axis for tensor tomography to determine the clay mineral orientation in shale, we demonstrate the approach on a sample of bone, measured using two tomography axes. The primary constituent of bone is the mineralized collagen fibre matrix, where the mineralized phase consists of hydroxyapatite (HA) crystallites (Stock, 2015), located within and outside the collagen fibre bundles. The textured wide-angle X-ray scattering from bone originates primarily from the electron density periodicity within the HA crystallites, and the preferred orientation of the crystallites with respect to the trabecula gives information about the collagen fibre orientation, which is closely tied to the mechanical properties of bone (Wenk & Heidelbach, 1999; Stock, 2015).

We have in a previous study used XRDTT to determine the 3D *c*-axis orientation of bone mineral hydroxyapatite crystallites of the same sample (Mürer *et al.*, 2021). Measurements of the bone and cartilage sample were done by a tensorial tomography measurement scheme utilizing two sample rotation axes (Liebi *et al.*, 2018). α denotes the fast-axis *tomography* rotation and β the tilt axis. $\beta = 0^\circ$ corresponds to single-axis tomography, as was used for the shale sample. For $\beta = 0^\circ$, 61 projections were recorded with $\Delta\alpha = 3.0^\circ$, thus giving $\alpha \in [0^\circ, 180^\circ]$. For $\beta \in [5^\circ, 40^\circ]$, i.e. the additional sample tilts for tensor tomography, $\Delta\alpha = 180^\circ / (60^\circ \cdot \cos(\beta))$ was used with $\alpha \in [0^\circ, 360^\circ]$. The factor $\cos(\beta)$ accounts for the distribution of equally spaced sampling points on a sphere (Liebi *et al.*, 2018). To collect each projection, the sample was raster scanned in (x,y) with 65 × 67 steps. The scan in (x,y) was repeated for 259 different projections, giving a total number of $65 \times 67 \times 259 \approx 1.1 \cdot 10^6$ recorded diffraction patterns. The exposure time for each diffraction pattern was 50 ms. The total measurement time for the bone-cartilage sample was approximately 26 hours, including overhead time for sample translation and rotation.

The femorotibial bone-cartilage joint sample was cut from a hindlimb femoral condyle of a 60-day-old piglet. No animal was euthanized specifically for this study, and the hindlimb originated from a previous study approved by the Norwegian Animal Research Authority. The whole hindlimb was formalin fixed, stored in a 70 wt.% ethanol/water solution, and the extracted section surrounding the bone-cartilage interface at the medial condyle of dimensions ~$2.7 \times 2.1 \times 2.1$ mm^3 was mounted inside a 3 mm polyimide tube, ensuring that he sample stayed immersed in ethanol-water solution, and thus hydrated, both before and during the experiment.

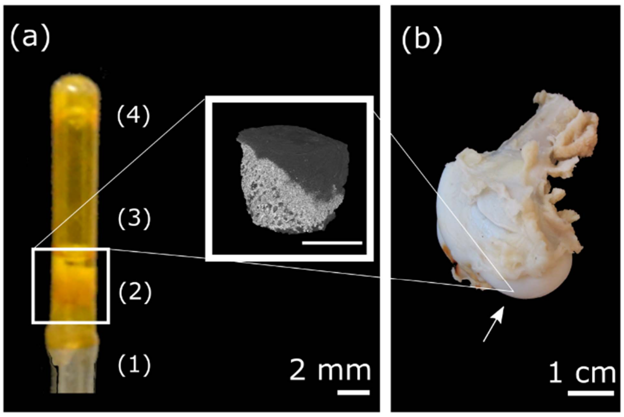

Figure S7.1 Sample preparation of bone and cartilage sample for XRDTT. (a) Sample holder used to keep the sample hydrated during the experiments. (1) Steel rod; (2) Sample mounted on rubber plug; (3) Polyimide ("Kapton") tube; (4) Epoxy droplet. (b) Distal end of a femur (hind-limb) of a young pig. The location where the studied sample was extracted from is marked by a white arrow.

An overview of the bone-cartilage sample is presented in Fig. S7.1. As shown in the PPC-CT tomograms in Fig. S7.2a and S7.2b, the highly scattering and attenuating bone minerals in the sample appear as high intensity (bright) regions. A diffraction pattern obtained with the X-ray beam going through the center of the sample is shown in Fig. S7.2c, with the corresponding azimuthally averaged diffractogram provided in Fig. S7.2d. All observed Bragg peaks in Fig. S7.2d originate from HA (Hughes *et al.*, 1989). The HA002 and HA004 peaks displayed texture, apparent as azimuthal intensity variations in Fig. S7.2e, consistent with a preferred orientation of the HA *c*-axis(Wenk & Heidelbach, 1999). Texture could not be seen in the other Debye-Scherrer rings, because of the many overlapping Bragg peaks, consistent with previous reports from bone (Meneghini *et al.*, 2003). There was no HA scattering originating from the cartilage, with the notable exception of the mineralized cartilage zone close to the bone-cartilage interface. Fig. S7.2f shows the dominant scattering direction of the HA002 peak obtained for a randomly chosen projection with $(\alpha, \beta) = (60^{\circ}, 0^{\circ})$, with each pixel (x,y) in Fig. S7.2f corresponding to a single measured diffraction pattern, all revealing HA002 scattering in approximately the same direction perpendicular to the bone-cartilage interface. The irregularities at the bottom and right edge are due to artefacts introduced by cutting the brittle bone with a surgical blade, as confirmed by comparison with the PPC-CT tomograms.

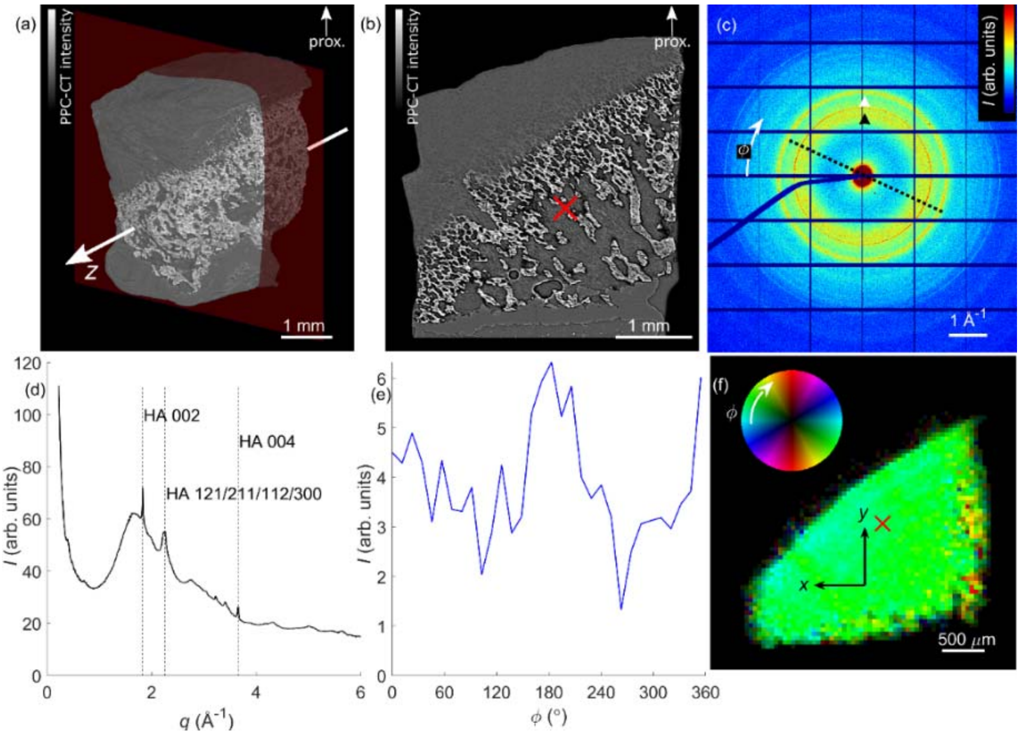

Figure S7.2 Scattering from the bone-cartilage sample. (a) 3D PPC-CT tomogram, with the proximal direction indicated. (b) PPC-CT cross section corresponding to the plane indicated in (a). (c) Representative diffraction pattern (on linear intensity scale) from the bone region of the sample. The black and white arrow heads indicate the HA002 and the overlapping HA121/211/112/300 Bragg peaks, respectively. The dotted line indicates the dominant scattering direction for the HA002 peak. (d) Azimuthal average of the diffraction pattern in (c). (e) Azimuthal intensity variations of the HA002 scattering, revealing a preferred orientation of the HA crystallites, obtained by integrating the measured intensity over $q \in [1.77 \text{ Å}^{-1}, 1.88 \text{ Å}^{-1}]$ and subtracting the background scattering. (f) The dominating HA002 scattering direction. Each pixel corresponds to a single measured diffraction pattern. The location of the obtained diffraction pattern in (c) is indicated by a red cross.

Based on the anisotropy of the HA002 Bragg peak we used XRDTT to reconstruct the HA crystallite *c*-axis orientation in the bone-cartilage sample. The XRDTT reconstructions were based on (i) all 259 measured projections, and (ii) only the 61 projections obtained with $\beta = 0°$, corresponding to the measurement scheme in conventional attenuation-contrast CT (Kak, Avinash C., Slaney, 1987) and single-axis XRD-CT (Harding *et al.*, 1987; Stock *et al.*, 2008; Kleuker *et al.*, 1998). 2D cross-sections of the reconstructed tomograms are shown in Fig. S7.3a and S.7b, demonstrating a close resemblance between the two reconstructions. A quantitative comparison of the reconstructed preferred orientation reconstructed using all tomography axes and only a single tomography axis was done by calculating the angular difference in orientation $\gamma(\mathbf{r'})$ reconstructed from the two datasets, defined as

$$\gamma(\mathbf{r}') = \arccos\left(\hat{\mathbf{u}}_{\mathrm{HA002}}^{n=259}(\mathbf{r}') \cdot \hat{\mathbf{u}}_{\mathrm{HA002}}^{n=61}(\mathbf{r}')\right), \quad (S7.1)$$

where $\hat{\mathbf{u}}(\mathbf{r}')$ are unity vectors indicating the HA crystallite *c*-axis preferred orientation direction in each sample voxel. To compare the reconstructed reciprocal maps, determined by the reconstructed $a_l^m(\mathbf{r'})$, we compared the reconstructed Hermans' parameter $S(\mathbf{r'})$ (cf. Eq. 4 in the main article) obtained from the different reconstructions by

$$\Delta S(\mathbf{r}') = S_{\mathrm{HA002}}^{n=259}(\mathbf{r}') - S_{\mathrm{HA002}}^{n=61}(\mathbf{r}') \quad (S7.2)$$

From the maps of $\gamma(\mathbf{r'})$ and $\Delta S(\mathbf{r'})$ we observed the difference of the XRDTT tomograms reconstructed from all tomography axes and one tomography axes to be mainly restricted to the sample edges and close to gaps between the trabecula in the sample, cf. Fig. S7.3c and S7.3d. The analysis demonstrates that at least for the present dataset, the local HA *c*-axis orientation can be reliably reconstructed using a single tomography axis.

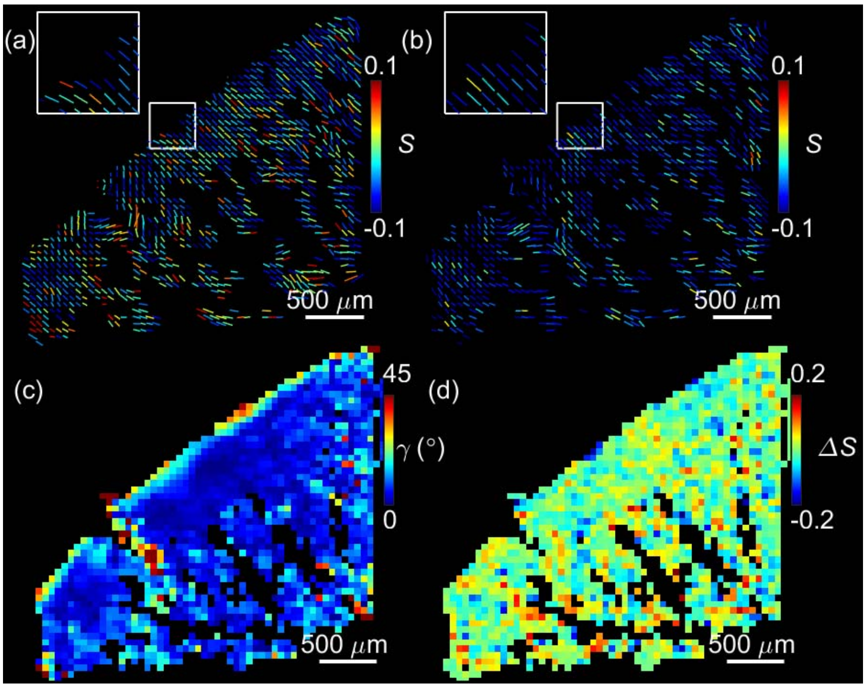

Figure S7.3 Comparison of HA orientation in bone for reconstructions based on 259 or 61 projections. (a) Cross section reconstructed using all 259 projections obtained with two tomography axes. (b) Cross-section reconstructed using only the 61 projections obtained for β = 0°. The color coding in (a) and (b) indicates the reconstructed Hermans' parameter *S*, cf. Eq. 4. (c) Angle between reconstructed preferred orientation directions in (a) and (b), cf. Eq. S7.1. (d) The difference in Hermans' parameter between (a) and (b), cf. Eq. S7.2.

S8. XRDTT reconstructions of clinochlore 001

By using the same strategy as for reconstruction of the clinochlore 002/kaolinite 002 Bragg peak as shown in Fig. 4 in the main article, XRDTT maps were generated using the broad clinochlore 001 peak, shown in Fig. S8. Similar orientation features were found as for the clinochlore 002/kaolinite 002 peak, although differing slightly due to Bragg peak overlap.

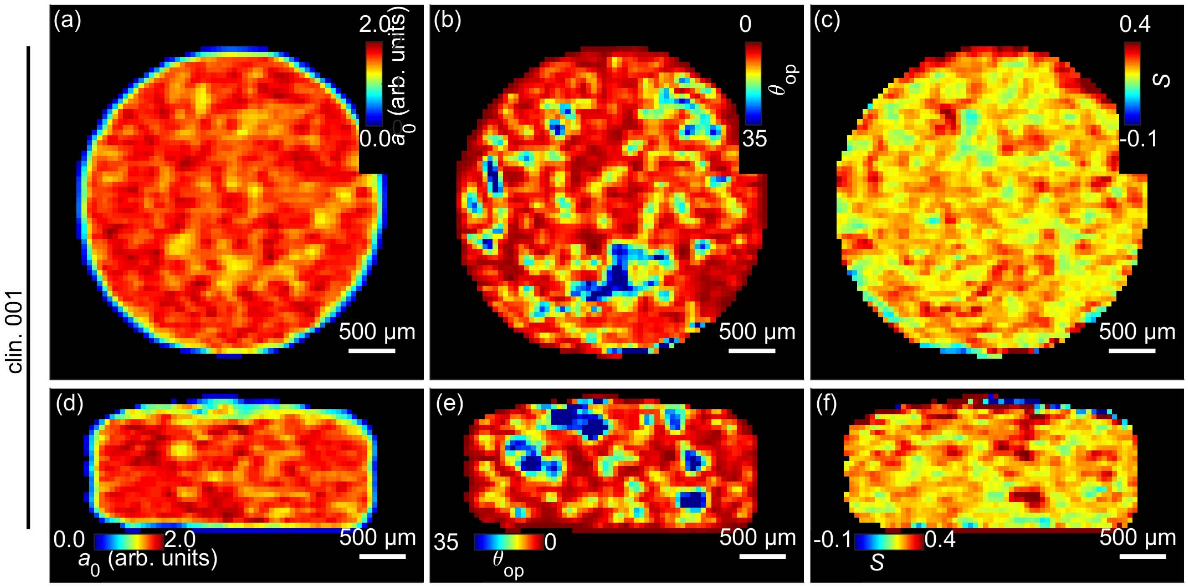

Figure S8 XRDTT reconstructions of clinochlore 001.

S9. Representative orientation distributions for clay minerals

Figure S9 shows the scattering resulting scattering intensity distributions reconstructed for two selected voxels of the sample. The scattering intensities are calculated by using Eq. 1.

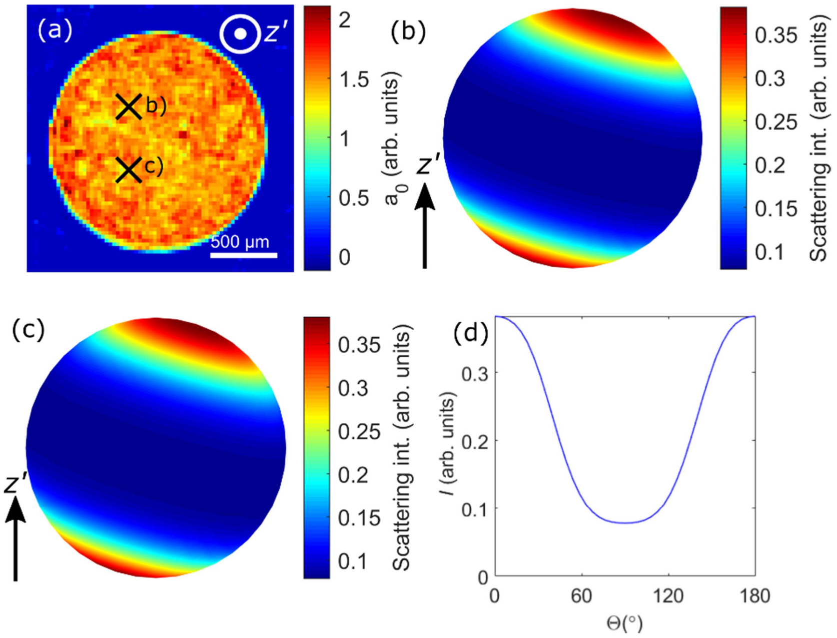

Figure S9 XRDTT cross-section and examples of reconstructed scattering intensity. a) a0 cross-section. b) Scattering distribution map from point marked "b)" in a). The reconstructed spherical coefficients and preferred orientation angles are a_0^0 = 1.37, a_2^0 = 0.39, a_4^0 = 0.02, a_6^0 = -0.03, θ_{op} = 19.0° and ϕ_{op} = 64.4°. c) Scattering distribution map from point marked "c)" in a). The reconstructed spherical coefficients and preferred orientation angles are a_0^0 = 1.43, a_2^0 = 0.40, a_4^0 = -0.09, a_6^0 = -0.03, θ_{op} = 5.1°. ϕ_{op} = 115.9°. d) Polar intensity distribution corresponding to b).

S10. Sinograms for multiple Bragg peaks

In the sample averaged diffraction patterns, we could identify 114 Bragg peaks between $q \in (0, 6.2)$ $Å^{-1}$. The generated sinograms for the cross-section corresponding to the cross-section in Fig. 6 are shown in Fig. S10.1-S10.4 for prominent peaks (cf. Fig. 2) for $q \in (0, 3.0)$ $Å^{-1}$. Peaks with

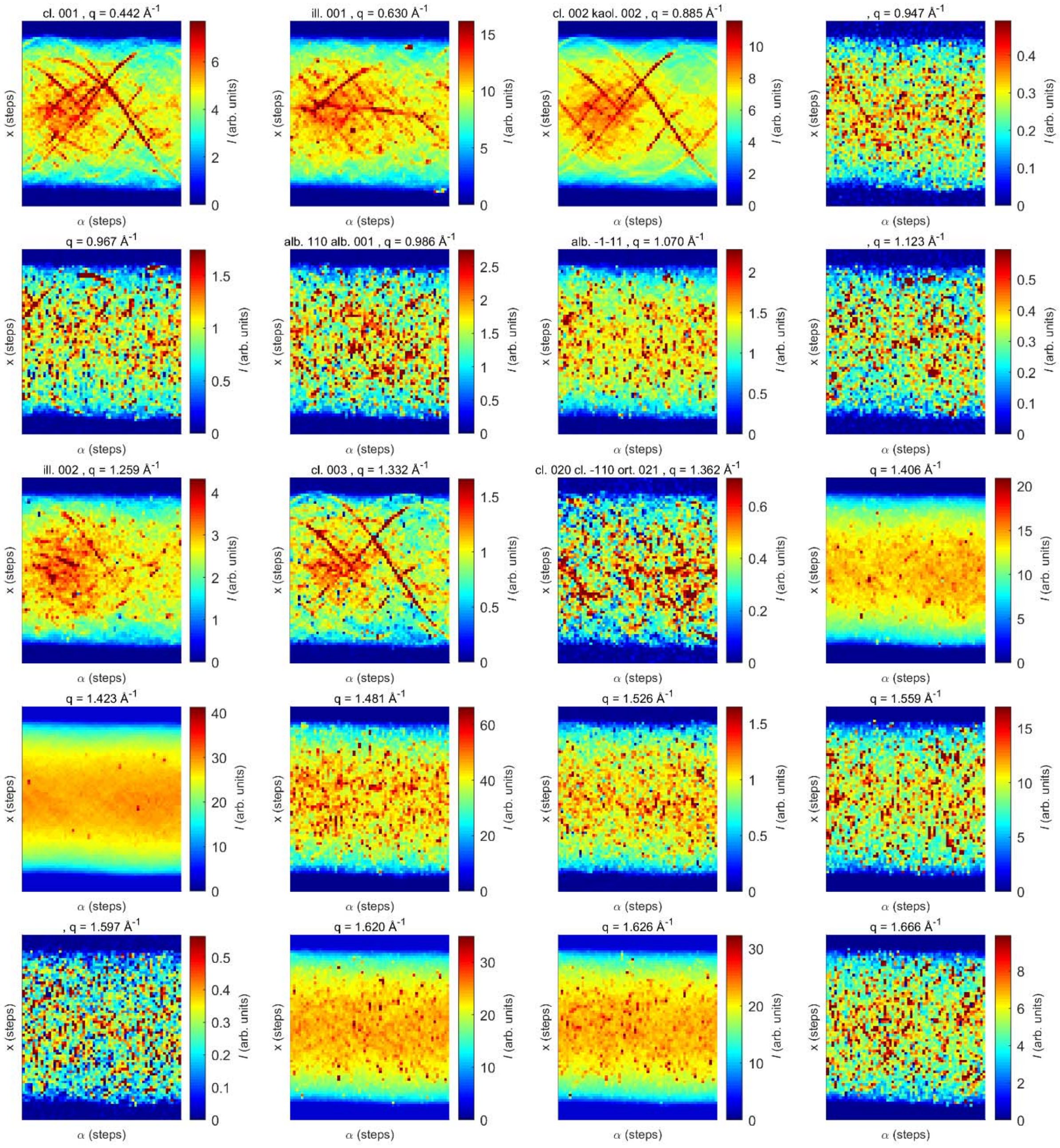

Figure S10.1 Parallel-beam XRD-CT sinograms for Bragg peaks in shale corresponding to a single cross-section. The different panels correspond to different q. Background has been subtracted by averaging the scattering intensity at the peak shoulders.

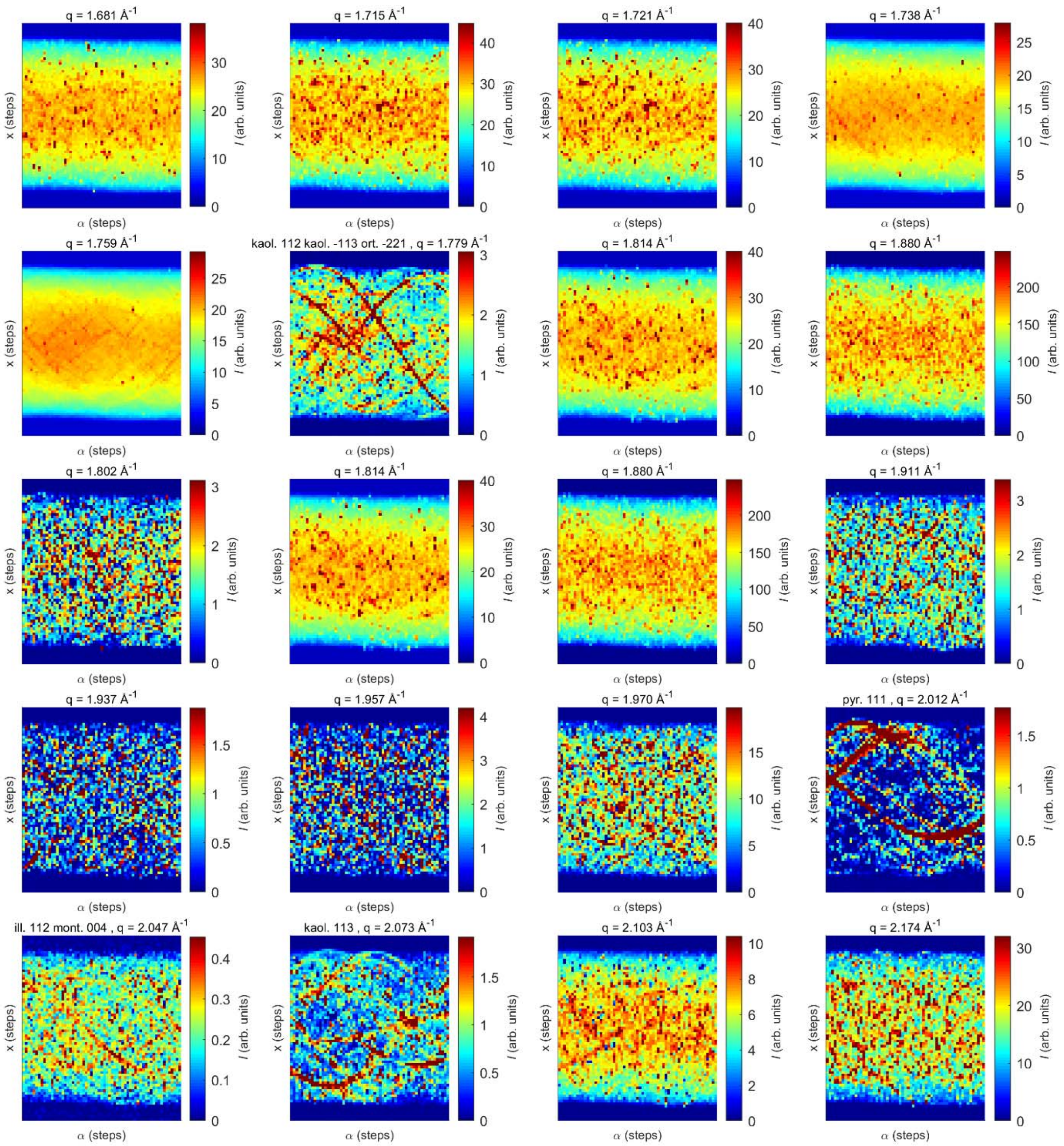

Figure S10.2 Parallel-beam XRD-CT sinograms for Bragg peaks in shale corresponding to a single cross-section. The different panels correspond to different q. Background has been subtracted by averaging the scattering intensity at the peak shoulders.

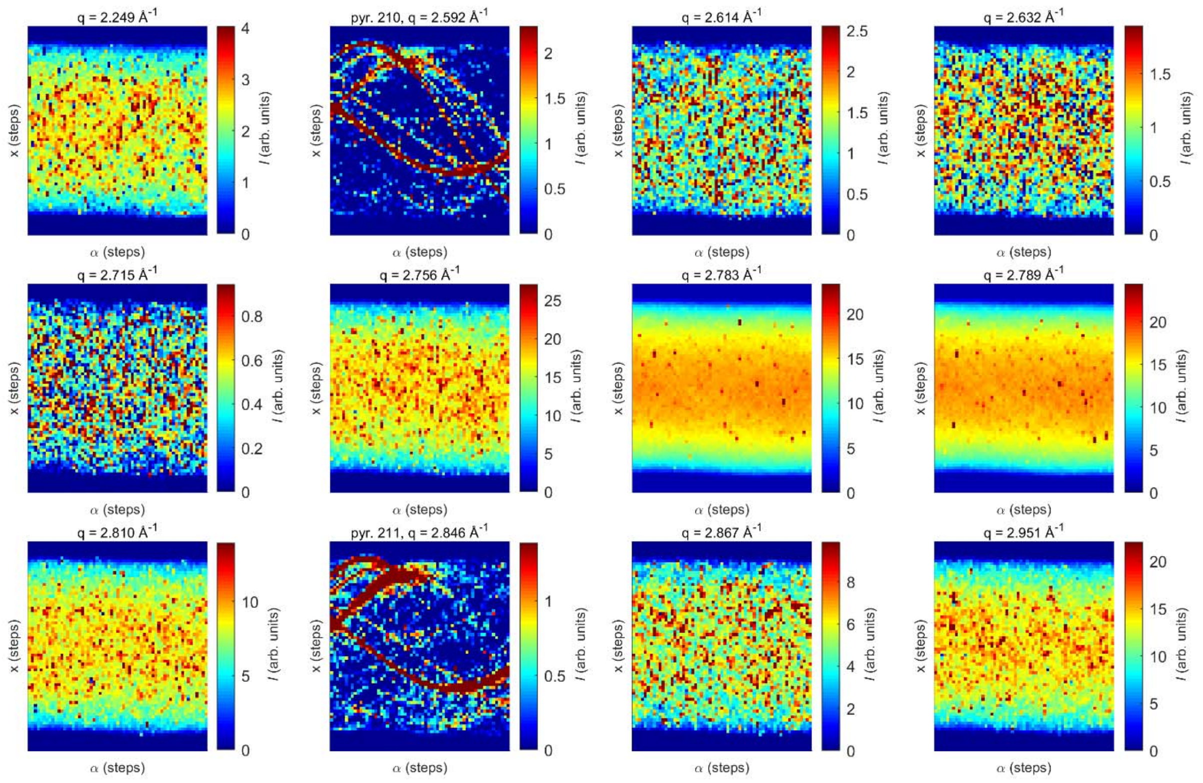

Figure S10.3 Parallel-beam XRD-CT sinograms for Bragg peaks in shale corresponding to a single cross-section. The different panels correspond to different q. Background has been subtracted by averaging the scattering intensity at the peak shoulders.

S11. Filtered backprojection reconstructed XRD-CT data

Isotropic XRD-CT reconstruction was done on sinograms $I(x,y,\alpha,q)$ separately for the integrated datasets of the shale sample without background subtraction, cf. 2.2. The unfiltered diffraction patterns were 1D median filtered in the azimuthal direction (in ϕ) before azimuthal averaging. XRD-CT reconstruction (Birkbak *et al.*, 2015) was done separately for each q (2048 radial bins). The reconstruction was done using the filtered backprojection (FBP) (Kak, Avinash C., Slaney, 1987) algorithm as implemented in the ASTRA toolbox (van Aarle *et al.*, 2016) in combination with custom-made macros. The reconstructed voxel size in XRD-CT was $(50\ \mu m)^3$. Several choices of filtering is available for reconstruction in isotropic XRD-CT. Figure S7 shows the comparison between median filtering the projections in φ before creating sinograms, or filtering the diffraction patterns in polar coordinates with an alpha-trimmed-mean filter with varying filter strength, before binning into radial bins, as previously demonstrated by (Vamvakeros *et al.*, 2015).

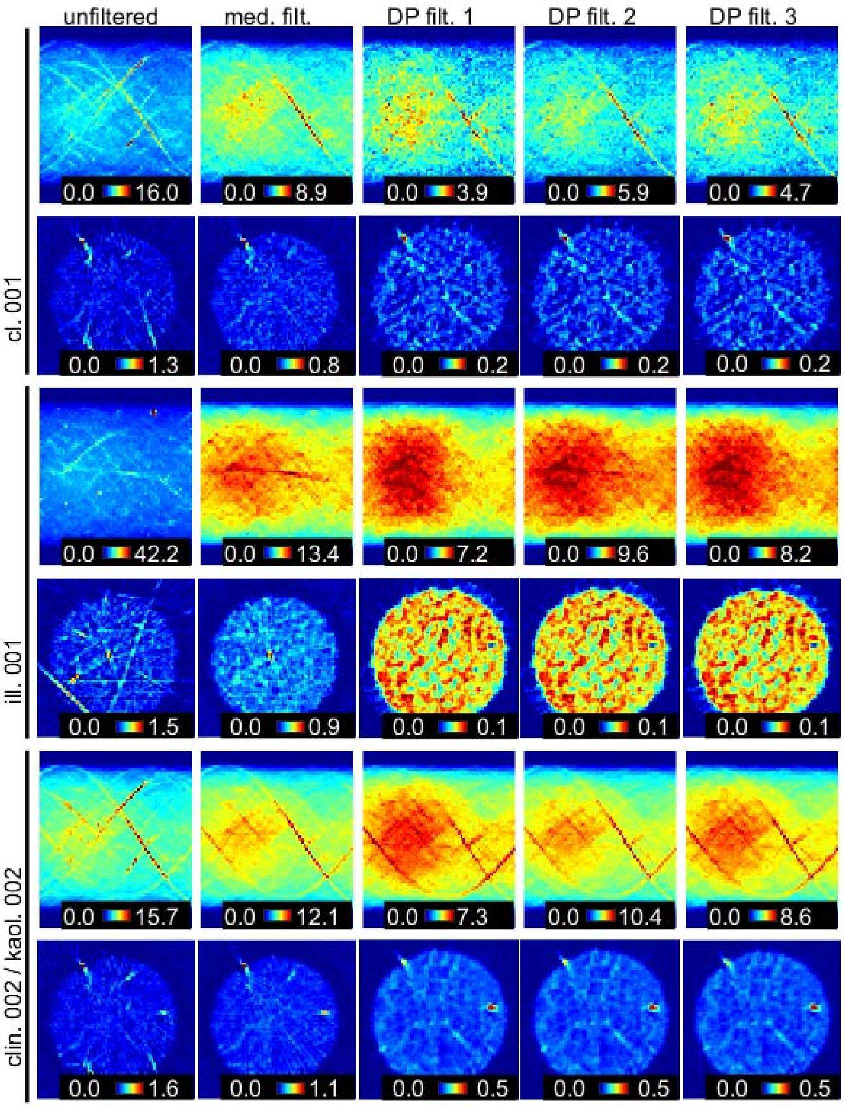

Figure S11.1 Different filtering strategies before XRD-CT reconstruction. "med. filt" denotes filtering of the azimuthal intensity distributions after radial and azimuthal integration, while "DP filt. 1", "DP filt. 2" and "DP filt. 3", are filtering of the diffraction patterns with different filter strengths (1-3) before radial and azimuthal integration.

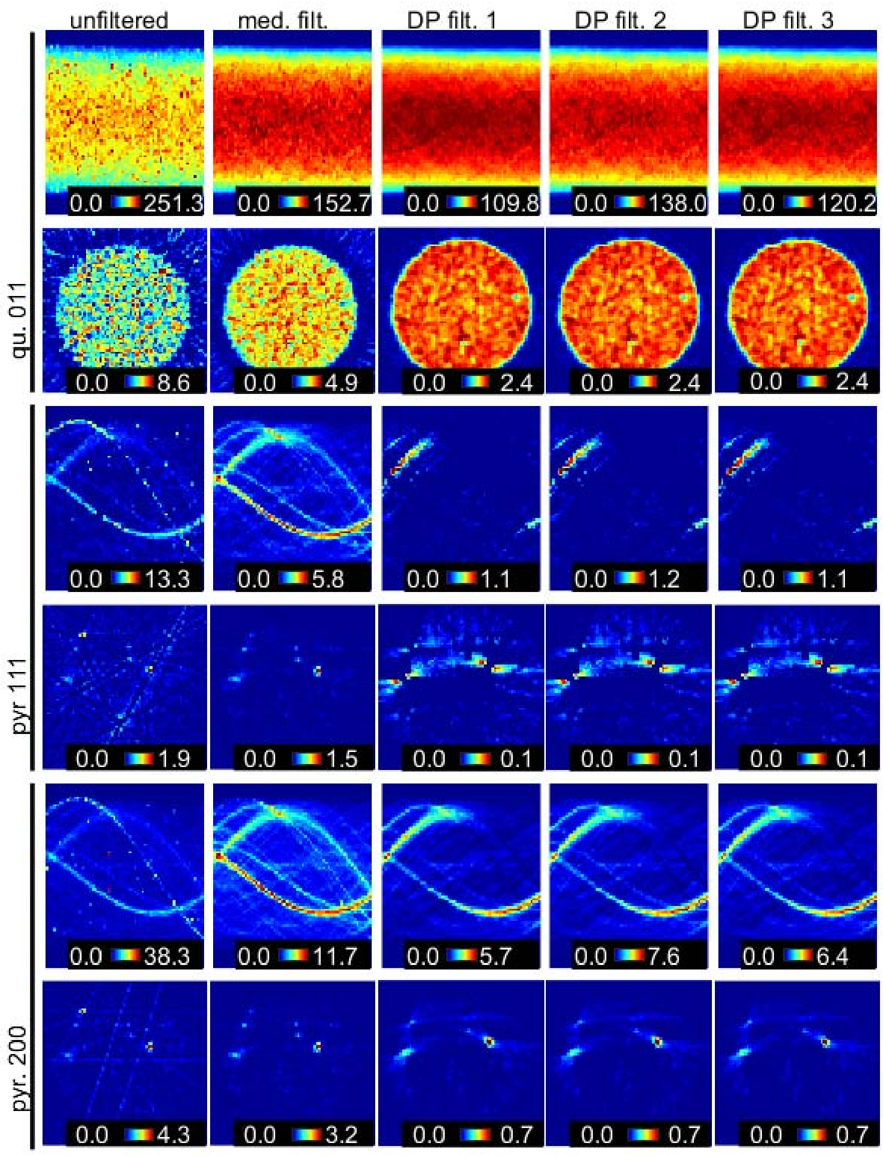

Figure S11.2 Different filtering strategies before XRD-CT reconstruction. “med. filt” denotes filtering of the azimuthal intensity distributions after radial and azimuthal integration, while “DP filt. 1”, “DP filt. 2” and “DP filt. 3”, are filtering of the diffraction patterns with different filter strengths (1-3) before radial and azimuthal integration.

S12. Reconstructed diffractograms

Figure S12 shows reconstructed diffractogram from the particles marked (1), (2) and (3) in the main article.

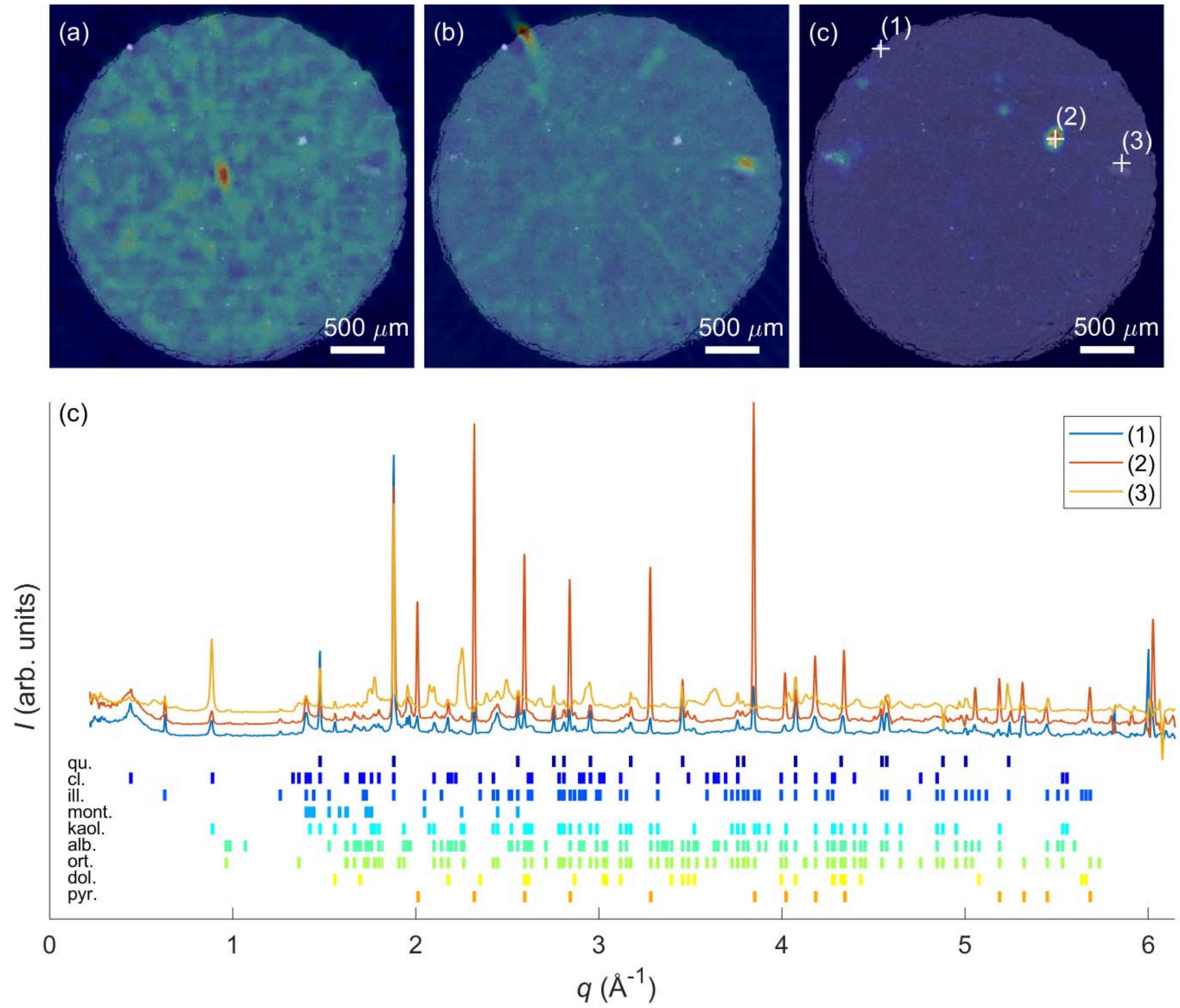

Figure S12 XRD-CT revealing the full diffractograms at localized sample points. (a-c) Overlay of attenuation-contrast cross-section in Fig 6a with (a) illite 002, (b) clinochlore 002/kaolinite 002 and (c) pyrite 200. (c) Reconstructed diffractograms for points (1), (2) and (3) indicated in (c), based on median filtered sinograms.

S13. XRD-CT and attenuation-contrast CT correlation analysis

A quantitative comparison of the attenuation-contrast CT and XRD-CT of Pierre shale was made by calculating the correlation between the attenuation-contrast sinograms and the XRD-CT sinograms for each q. This analysis gives similar information as for XRD-CT reconstruction, where local mineral content can be assigned to the different voxels, however without using XRD-CT reconstruction (see S12), which could lead to artefacts for large crystallites or oriented minerals. The correlation coefficient $r(q)$ is calculated by from the attenuation-contrast and XRD-CT sinograms by

$$r(q) = \frac{\sum_x \sum_\alpha \left(I_{\mathrm{att.CT}}(x,\alpha) - \bar{I}_{\mathrm{att.CT}}\right)\left(I_{\mathrm{XRD-CT}}(x,\alpha,q) - \bar{I}_{\mathrm{XRD-CT}}(q)\right)}{\sqrt{\sum_x \sum_\alpha \left(I_{\mathrm{att.CT}}(x,\alpha) - \bar{I}_{\mathrm{att.CT}}\right)^2}\ \sqrt{\sum_x \sum_\alpha \left(I_{\mathrm{XRD-CT}}(x,\alpha,q) - \bar{I}_{\mathrm{XRD-CT}}(q)\right)^2}}, \qquad \text{(S12.1)}$$

where

$$\bar{I}_{\mathrm{att.CT}} = \frac{1}{N_x N_\alpha} \sum_x \sum_\alpha I_{\mathrm{att.CT}}(x,\alpha) \qquad \text{(S12.2)}$$

and

$$\bar{I}_{\mathrm{XRD-CT}}(q) = \frac{1}{N_x N_\alpha} \sum_x \sum_\alpha I_{\mathrm{XRD-CT}}(x,\alpha,q). \qquad \text{(S12.3)}$$

The analysis gave high correlation between the attenuation-contrast sinogram curves for particles marked (1) and (2) for q corresponding to pyrite. Low correlation between the clinochlore-containing regions was observed, due to the lower signal to noise ratio of these sinograms.

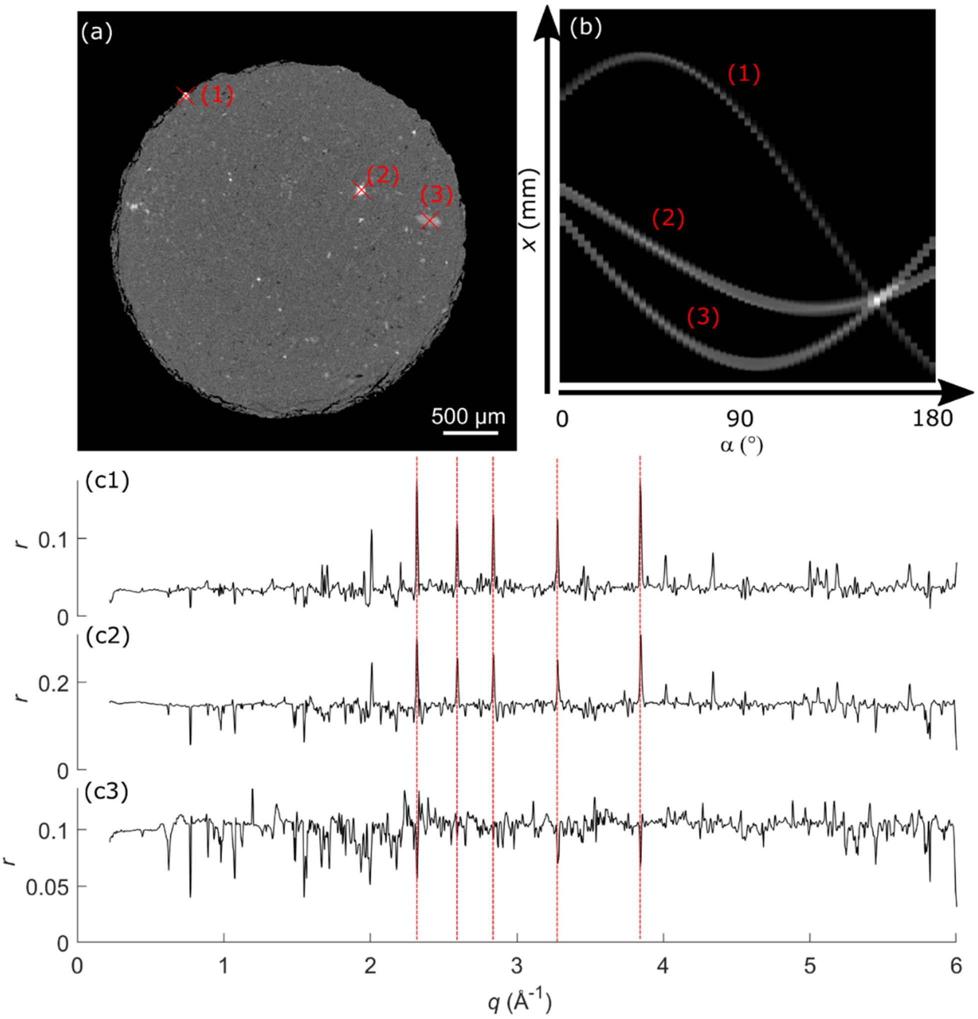

Figure S13 Correlation analysis between attenuation-contrast CT and XRD-CT of Pierre shale. (a) Measured cross-section. Particles are indicated with (1), (2) and (3). (b) Corresponding parallel beam sinogram (Radon transform) of points marked with (X) in (a). (c1-c3) Sinogram correlation between attenuation-contrast CT and XRD-CT. The red vertical lines indicate *q* corresponding to pyrite Bragg peaks.

References

van Aarle, W., Palenstijn, W. J., Cant, J., Janssens, E., Bleichrodt, F., Dabravolski, A., De Beenhouwer, J., Joost Batenburg, K. & Sijbers, J. (2016). *Opt. Express*. **24**, 25129.

Birkbak, M. E., Leemreize, H., Frølich, S., Stock, S. R. & Birkedal, H. (2015). *Nanoscale*. **7**, 18402–18410.

Harding, G., Kosanetzky, J. & Neitzel, U. (1987). *Med. Phys.* **515**, 515–525.

Hughes, J. M., Cameron, M. & Crowley, K. D. (1989). *Am. Mineral.* **74**, 870–876.

Kak, Avinash C., Slaney, M. (1987). Principles of Computerized Tomographic Imaging Society for Industrial and Applied Mathematics.

Kleuker, U., Suortti, P., W., W. & Spanne, P. (1998). *Phys. Med. Biol.* **43**, 2911–2923.

Liebi, M., Georgiadis, M., Kohlbrecher, J., Holler, M., Raabe, J., Usov, I., Menzel, A., Schneider, P., Bunk, O. & Guizar-Sicairos, M. (2018). *Acta Crystallogr. Sect. A Found. Adv.* **74**, 12–24.

Meneghini, C., Dalconi, M. C., Nuzzo, S., Mobilio, S. & Wenk, R. H. (2003). *Biophys. J.* **84**, 2021–2029.

Mürer, F. K., Chattopadhyay, B., Madathiparambil, A. S., Tekseth, K. R., Di Michiel, M., Liebi, M., Lilledahl, M. B., Olstad, K. & Breiby, D. W. (2021). *Sci. Rep.* **11**, 1–12.

Stock, S. R. (2015). *Calcif. Tissue Int.* **97**, 262–280.

Stock, S. R., De Carlo, F. & Almer, J. D. (2008). *J. Struct. Biol.* **161**, 144–150.

Vamvakeros, A., Jacques, S. D. M., Di Michiel, M., Middelkoop, V., Egan, C. K., Cernik, R. J. & Beale, A. M. (2015). *J. Appl. Crystallogr.* **48**, 1943–1955.

Wenk, H. R. & Heidelbach, F. (1999). *Bone*. **24**, 361–369.
